# Supplementary figures and images for: Distinct roles of nonmuscle myosin II isoforms for establishing tension and elasticity during cell morphodynamics
Source: eLife. 2021 Aug 10;10:e71888. doi: 10.7554/eLife.71888 (PMC8391736; doi:10.7554/eLife.71888)

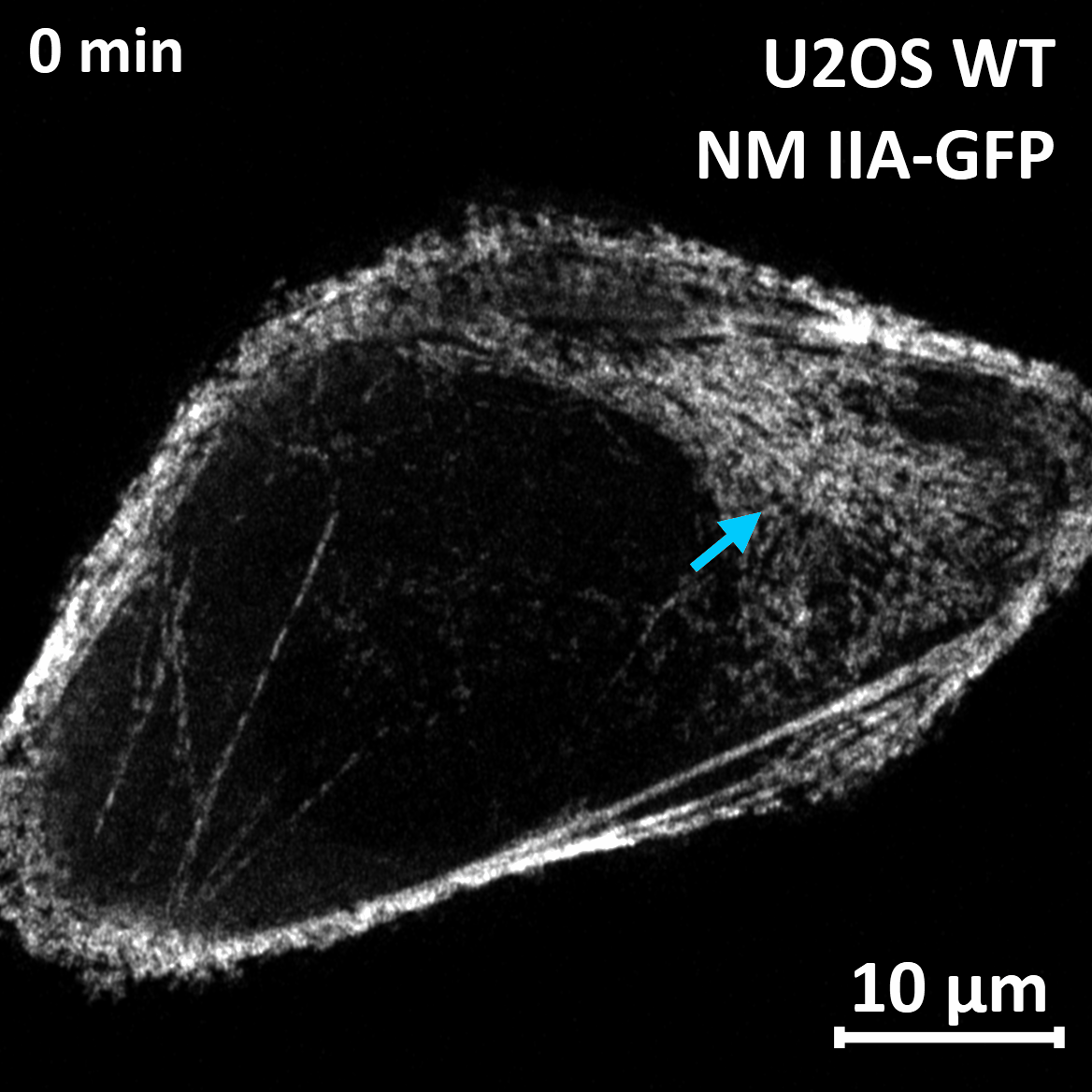

Supplement: Supplementary file 1 [file elife-71888-fig4-video1.gif]

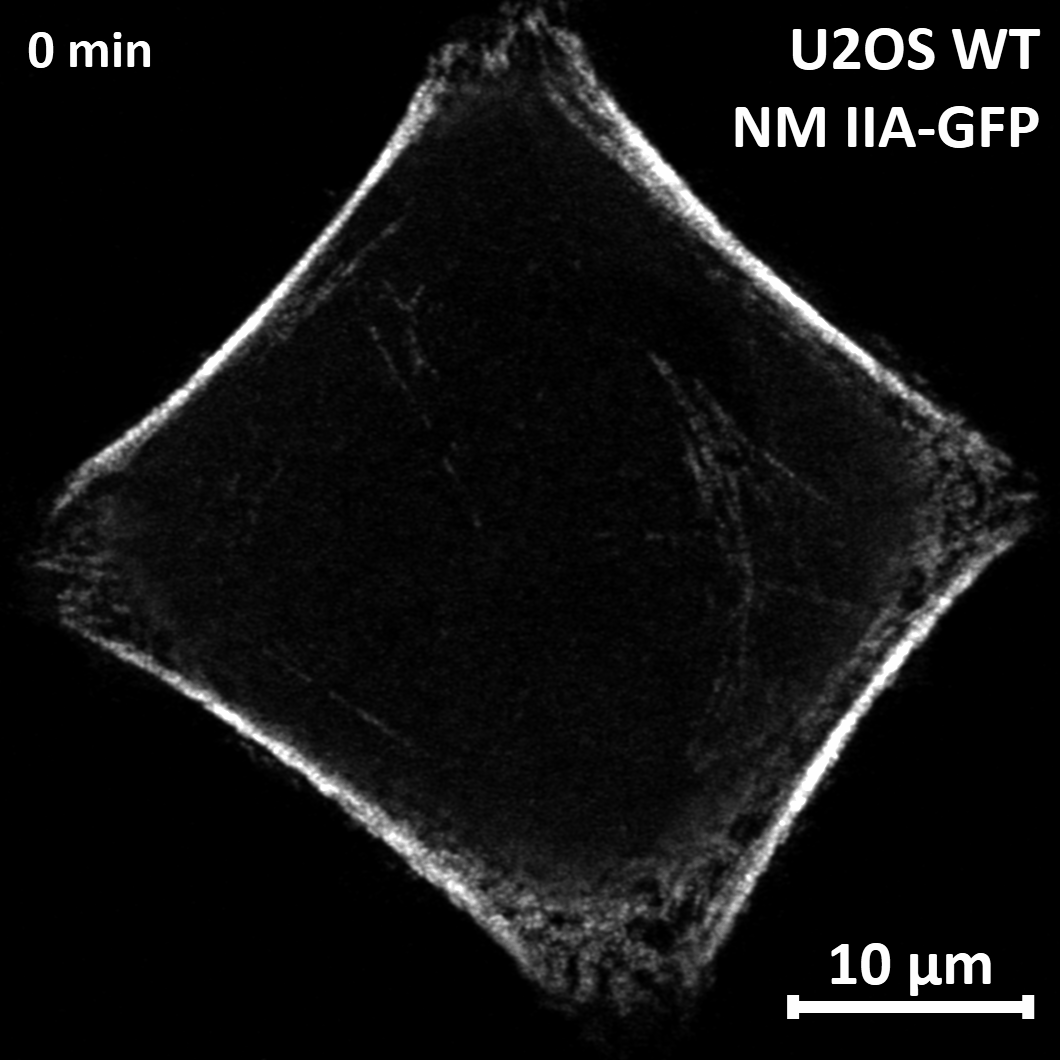

Supplement: Supplementary file 2 [file elife-71888-fig4-video2.gif]

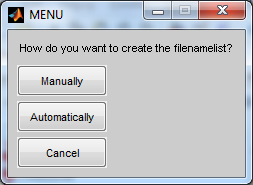

Supplement: Figure 5—source code 1. [file elife-71888-fig5-code1.zip › Source code/Digital Image Correlation/Correlation_Tracking_Guide_2010-Dateien/image001.png]

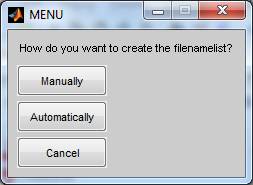

Supplement: Figure 5—source code 1. [file elife-71888-fig5-code1.zip › Source code/Digital Image Correlation/Correlation_Tracking_Guide_2010-Dateien/image002.jpg]

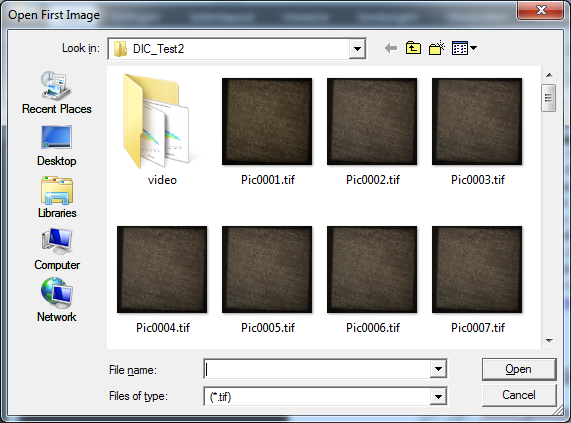

Supplement: Figure 5—source code 1. [file elife-71888-fig5-code1.zip › Source code/Digital Image Correlation/Correlation_Tracking_Guide_2010-Dateien/image003.png]

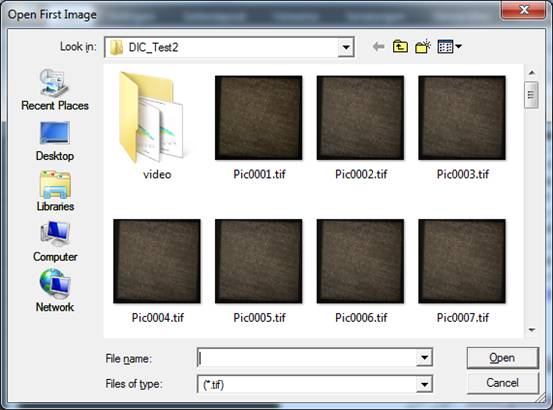

Supplement: Figure 5—source code 1. [file elife-71888-fig5-code1.zip › Source code/Digital Image Correlation/Correlation_Tracking_Guide_2010-Dateien/image004.jpg]

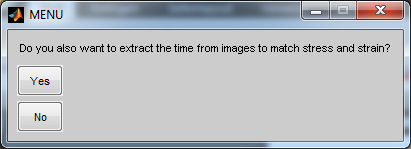

Supplement: Figure 5—source code 1. [file elife-71888-fig5-code1.zip › Source code/Digital Image Correlation/Correlation_Tracking_Guide_2010-Dateien/image005.png]

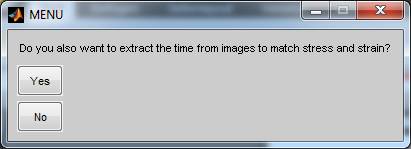

Supplement: Figure 5—source code 1. [file elife-71888-fig5-code1.zip › Source code/Digital Image Correlation/Correlation_Tracking_Guide_2010-Dateien/image006.jpg]

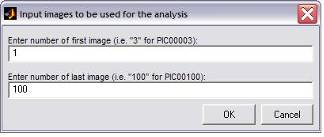

Supplement: Figure 5—source code 1. [file elife-71888-fig5-code1.zip › Source code/Digital Image Correlation/Correlation_Tracking_Guide_2010-Dateien/image007.jpg]

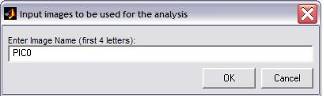

Supplement: Figure 5—source code 1. [file elife-71888-fig5-code1.zip › Source code/Digital Image Correlation/Correlation_Tracking_Guide_2010-Dateien/image008.jpg]

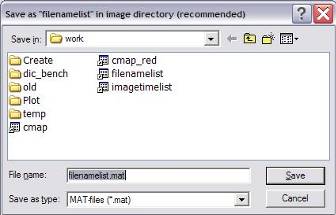

Supplement: Figure 5—source code 1. [file elife-71888-fig5-code1.zip › Source code/Digital Image Correlation/Correlation_Tracking_Guide_2010-Dateien/image009.jpg]

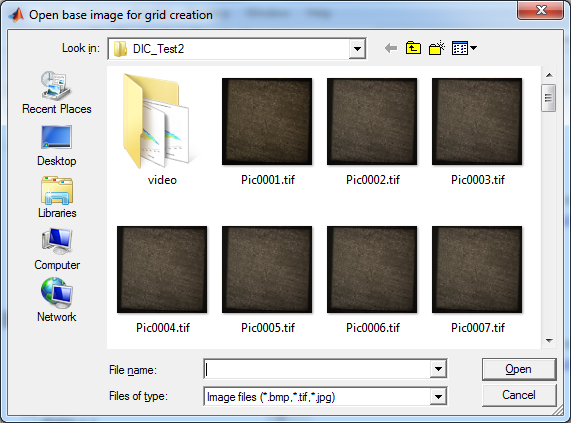

Supplement: Figure 5—source code 1. [file elife-71888-fig5-code1.zip › Source code/Digital Image Correlation/Correlation_Tracking_Guide_2010-Dateien/image010.png]

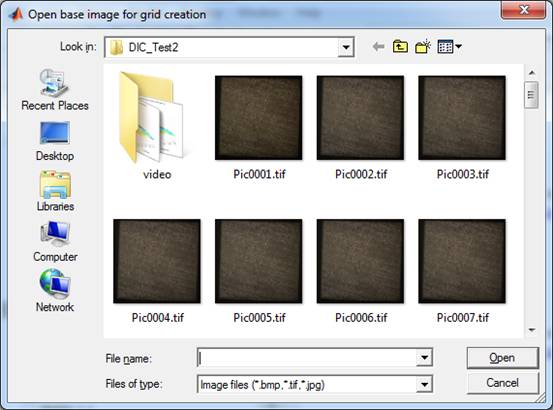

Supplement: Figure 5—source code 1. [file elife-71888-fig5-code1.zip › Source code/Digital Image Correlation/Correlation_Tracking_Guide_2010-Dateien/image011.jpg]

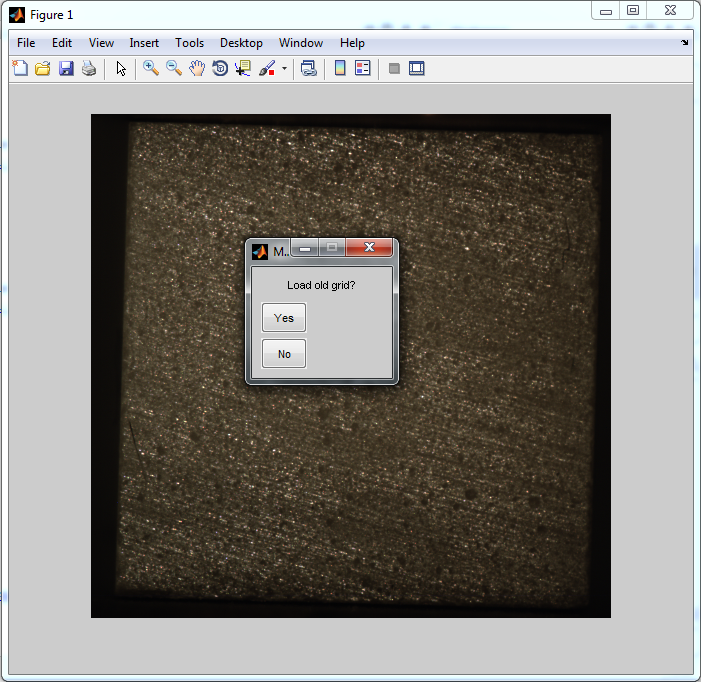

Supplement: Figure 5—source code 1. [file elife-71888-fig5-code1.zip › Source code/Digital Image Correlation/Correlation_Tracking_Guide_2010-Dateien/image012.png]

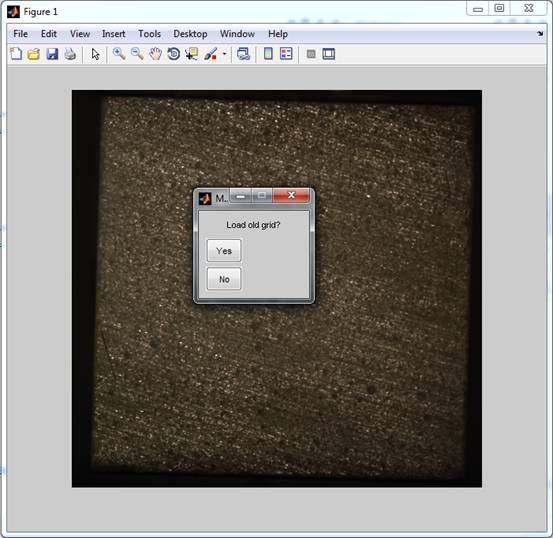

Supplement: Figure 5—source code 1. [file elife-71888-fig5-code1.zip › Source code/Digital Image Correlation/Correlation_Tracking_Guide_2010-Dateien/image013.jpg]

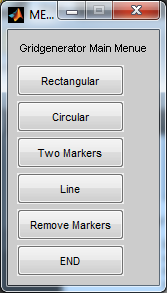

Supplement: Figure 5—source code 1. [file elife-71888-fig5-code1.zip › Source code/Digital Image Correlation/Correlation_Tracking_Guide_2010-Dateien/image014.png]

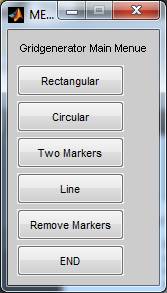

Supplement: Figure 5—source code 1. [file elife-71888-fig5-code1.zip › Source code/Digital Image Correlation/Correlation_Tracking_Guide_2010-Dateien/image015.jpg]

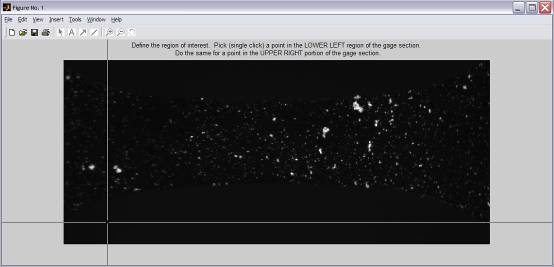

Supplement: Figure 5—source code 1. [file elife-71888-fig5-code1.zip › Source code/Digital Image Correlation/Correlation_Tracking_Guide_2010-Dateien/image016.jpg]

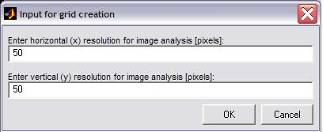

Supplement: Figure 5—source code 1. [file elife-71888-fig5-code1.zip › Source code/Digital Image Correlation/Correlation_Tracking_Guide_2010-Dateien/image017.jpg]

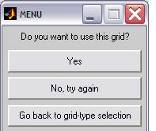

Supplement: Figure 5—source code 1. [file elife-71888-fig5-code1.zip › Source code/Digital Image Correlation/Correlation_Tracking_Guide_2010-Dateien/image018.jpg]

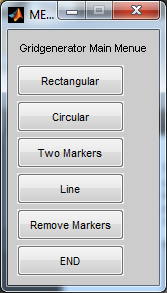

Supplement: Figure 5—source code 1. [file elife-71888-fig5-code1.zip › Source code/Digital Image Correlation/Correlation_Tracking_Guide_2010-Dateien/image019.png]

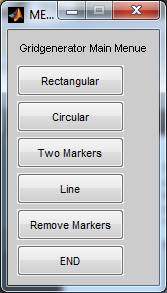

Supplement: Figure 5—source code 1. [file elife-71888-fig5-code1.zip › Source code/Digital Image Correlation/Correlation_Tracking_Guide_2010-Dateien/image020.jpg]

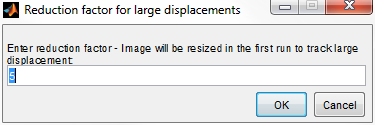

Supplement: Figure 5—source code 1. [file elife-71888-fig5-code1.zip › Source code/Digital Image Correlation/Correlation_Tracking_Guide_2010-Dateien/image021.png]

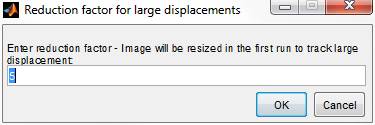

Supplement: Figure 5—source code 1. [file elife-71888-fig5-code1.zip › Source code/Digital Image Correlation/Correlation_Tracking_Guide_2010-Dateien/image022.jpg]

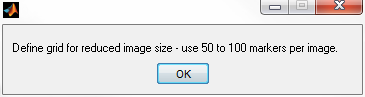

Supplement: Figure 5—source code 1. [file elife-71888-fig5-code1.zip › Source code/Digital Image Correlation/Correlation_Tracking_Guide_2010-Dateien/image023.png]

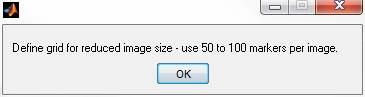

Supplement: Figure 5—source code 1. [file elife-71888-fig5-code1.zip › Source code/Digital Image Correlation/Correlation_Tracking_Guide_2010-Dateien/image024.jpg]

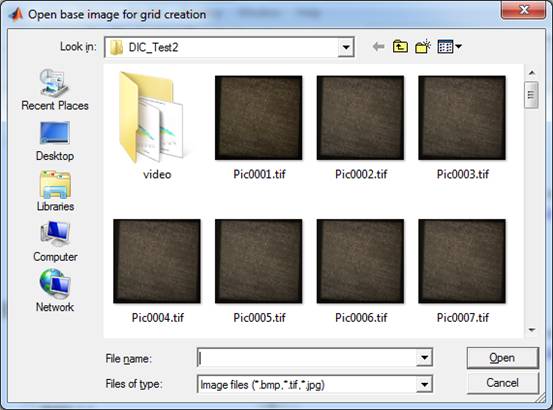

Supplement: Figure 5—source code 1. [file elife-71888-fig5-code1.zip › Source code/Digital Image Correlation/Correlation_Tracking_Guide_2010-Dateien/image025.jpg]

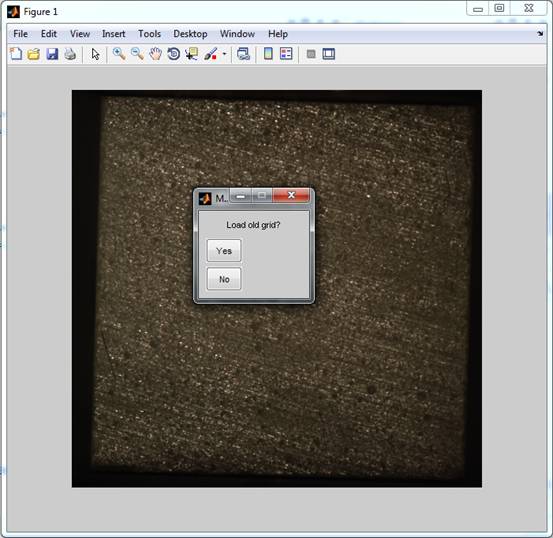

Supplement: Figure 5—source code 1. [file elife-71888-fig5-code1.zip › Source code/Digital Image Correlation/Correlation_Tracking_Guide_2010-Dateien/image026.jpg]

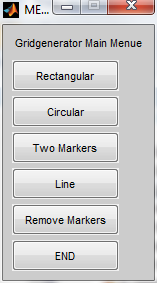

Supplement: Figure 5—source code 1. [file elife-71888-fig5-code1.zip › Source code/Digital Image Correlation/Correlation_Tracking_Guide_2010-Dateien/image027.png]

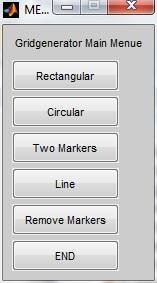

Supplement: Figure 5—source code 1. [file elife-71888-fig5-code1.zip › Source code/Digital Image Correlation/Correlation_Tracking_Guide_2010-Dateien/image028.jpg]

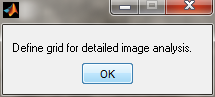

Supplement: Figure 5—source code 1. [file elife-71888-fig5-code1.zip › Source code/Digital Image Correlation/Correlation_Tracking_Guide_2010-Dateien/image029.png]

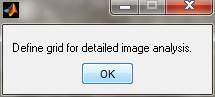

Supplement: Figure 5—source code 1. [file elife-71888-fig5-code1.zip › Source code/Digital Image Correlation/Correlation_Tracking_Guide_2010-Dateien/image030.jpg]

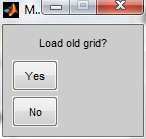

Supplement: Figure 5—source code 1. [file elife-71888-fig5-code1.zip › Source code/Digital Image Correlation/Correlation_Tracking_Guide_2010-Dateien/image031.png]

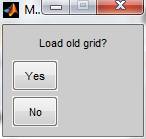

Supplement: Figure 5—source code 1. [file elife-71888-fig5-code1.zip › Source code/Digital Image Correlation/Correlation_Tracking_Guide_2010-Dateien/image032.jpg]

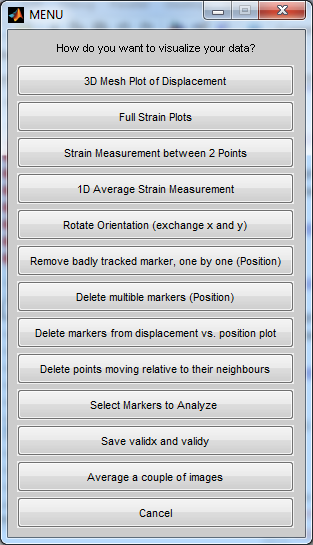

Supplement: Figure 5—source code 1. [file elife-71888-fig5-code1.zip › Source code/Digital Image Correlation/Correlation_Tracking_Guide_2010-Dateien/image033.png]

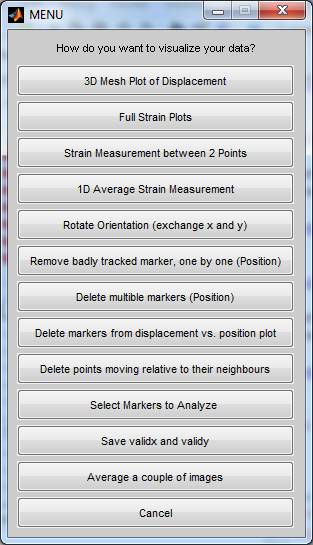

Supplement: Figure 5—source code 1. [file elife-71888-fig5-code1.zip › Source code/Digital Image Correlation/Correlation_Tracking_Guide_2010-Dateien/image034.jpg]

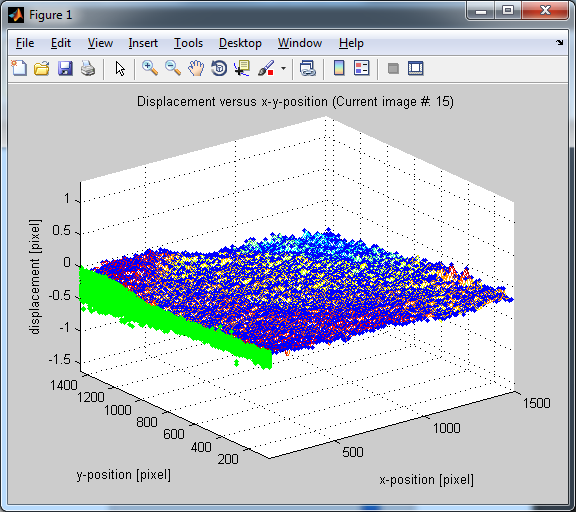

Supplement: Figure 5—source code 1. [file elife-71888-fig5-code1.zip › Source code/Digital Image Correlation/Correlation_Tracking_Guide_2010-Dateien/image035.png]

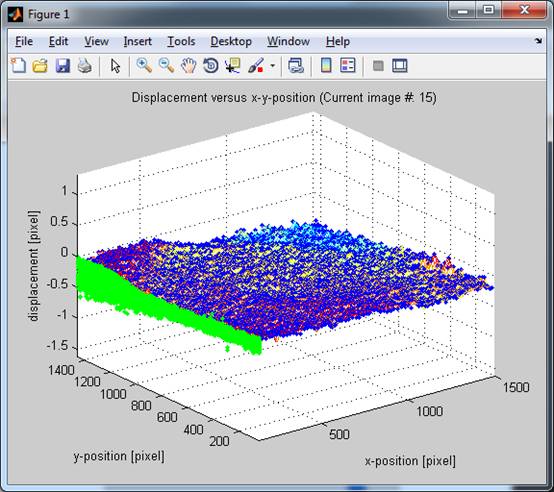

Supplement: Figure 5—source code 1. [file elife-71888-fig5-code1.zip › Source code/Digital Image Correlation/Correlation_Tracking_Guide_2010-Dateien/image036.jpg]

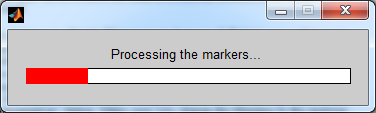

Supplement: Figure 5—source code 1. [file elife-71888-fig5-code1.zip › Source code/Digital Image Correlation/Correlation_Tracking_Guide_2010-Dateien/image037.png]

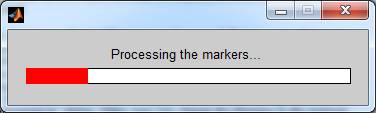

Supplement: Figure 5—source code 1. [file elife-71888-fig5-code1.zip › Source code/Digital Image Correlation/Correlation_Tracking_Guide_2010-Dateien/image038.jpg]

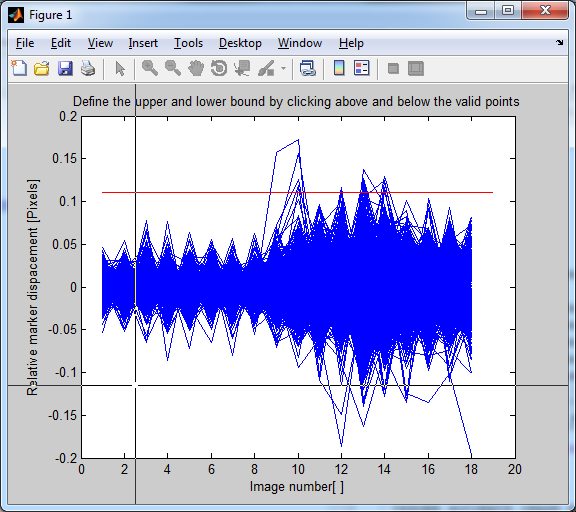

Supplement: Figure 5—source code 1. [file elife-71888-fig5-code1.zip › Source code/Digital Image Correlation/Correlation_Tracking_Guide_2010-Dateien/image039.png]

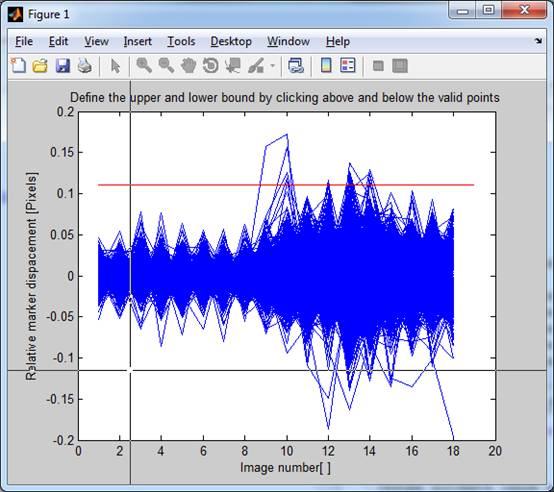

Supplement: Figure 5—source code 1. [file elife-71888-fig5-code1.zip › Source code/Digital Image Correlation/Correlation_Tracking_Guide_2010-Dateien/image040.jpg]

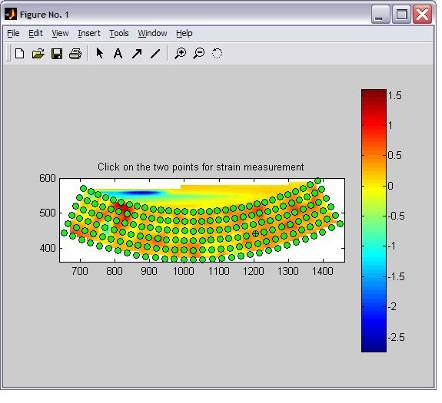

Supplement: Figure 5—source code 1. [file elife-71888-fig5-code1.zip › Source code/Digital Image Correlation/Correlation_Tracking_Guide_2010-Dateien/image041.jpg]

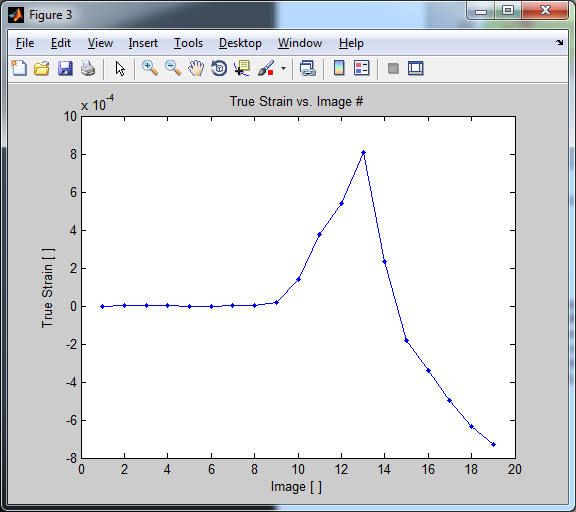

Supplement: Figure 5—source code 1. [file elife-71888-fig5-code1.zip › Source code/Digital Image Correlation/Correlation_Tracking_Guide_2010-Dateien/image042.png]

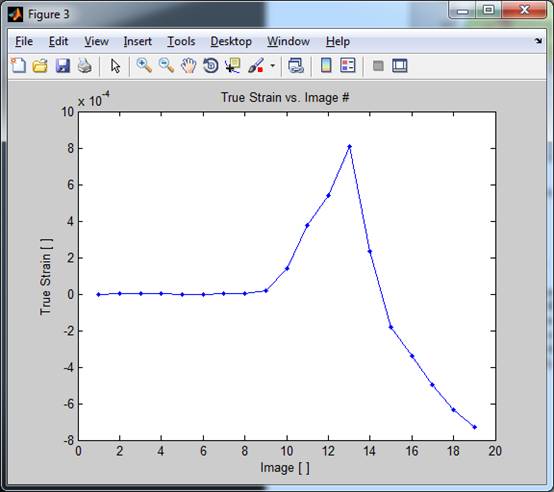

Supplement: Figure 5—source code 1. [file elife-71888-fig5-code1.zip › Source code/Digital Image Correlation/Correlation_Tracking_Guide_2010-Dateien/image043.jpg]

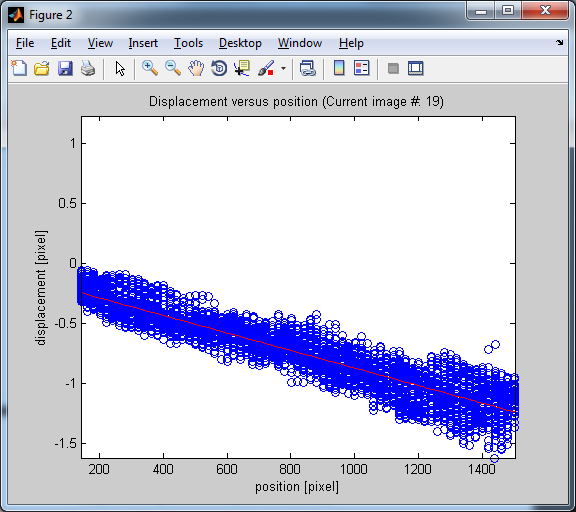

Supplement: Figure 5—source code 1. [file elife-71888-fig5-code1.zip › Source code/Digital Image Correlation/Correlation_Tracking_Guide_2010-Dateien/image044.png]

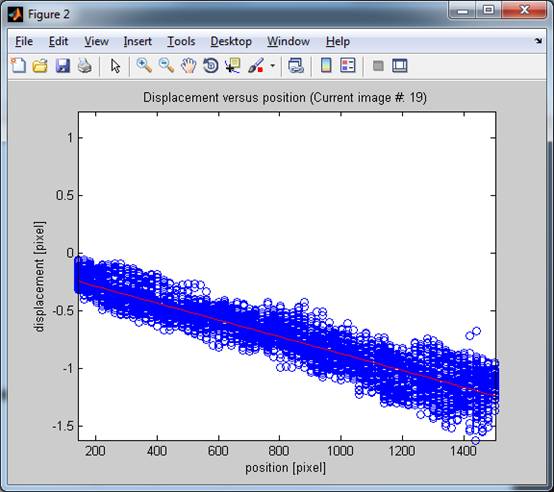

Supplement: Figure 5—source code 1. [file elife-71888-fig5-code1.zip › Source code/Digital Image Correlation/Correlation_Tracking_Guide_2010-Dateien/image045.jpg]

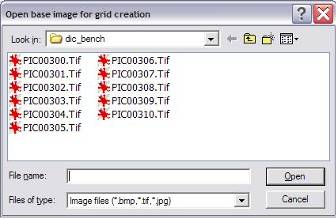

Supplement: Figure 5—source code 1. [file elife-71888-fig5-code1.zip › Source code/Digital Image Correlation/Correlation_Tracking_Guide_2010-Dateien/image046.jpg]

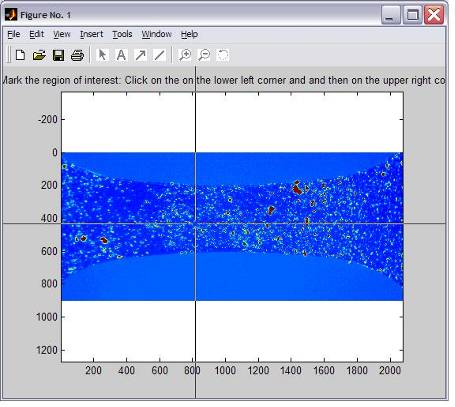

Supplement: Figure 5—source code 1. [file elife-71888-fig5-code1.zip › Source code/Digital Image Correlation/Correlation_Tracking_Guide_2010-Dateien/image047.jpg]

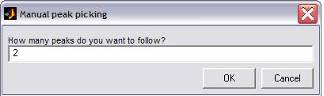

Supplement: Figure 5—source code 1. [file elife-71888-fig5-code1.zip › Source code/Digital Image Correlation/Correlation_Tracking_Guide_2010-Dateien/image048.jpg]

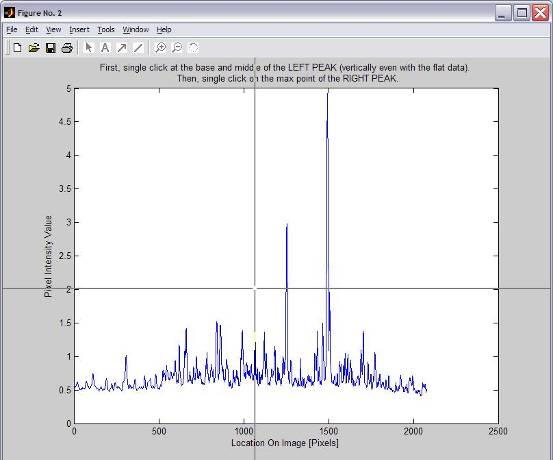

Supplement: Figure 5—source code 1. [file elife-71888-fig5-code1.zip › Source code/Digital Image Correlation/Correlation_Tracking_Guide_2010-Dateien/image049.jpg]

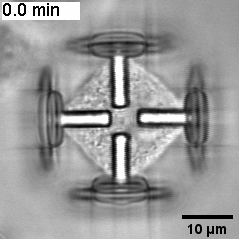

Supplement: Supplementary file 4 [file elife-71888-fig5-video1.gif]

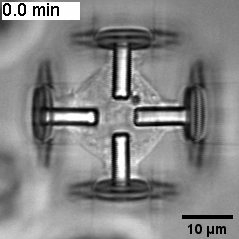

Supplement: Supplementary file 5 [file elife-71888-fig5-video2.gif]

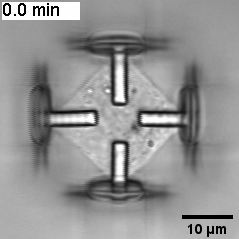

Supplement: Supplementary file 6 [file elife-71888-fig5-video3.gif]

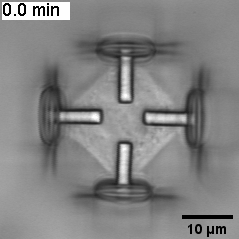

Supplement: Supplementary file 7 [file elife-71888-fig5-video4.gif]

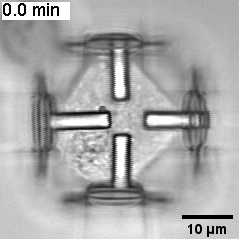

Supplement: Supplementary file 8 [file elife-71888-fig5-video5.gif]

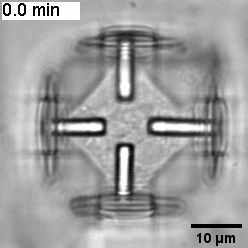

Supplement: Supplementary file 9 [file elife-71888-fig5-video6.gif]

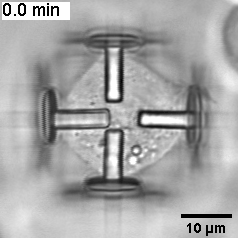

Supplement: Supplementary file 10 [file elife-71888-fig5-video7.gif]

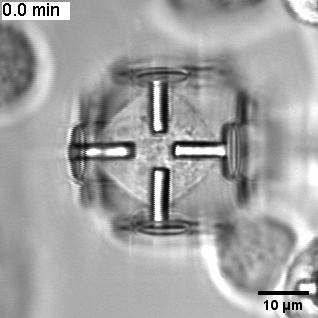

Supplement: Supplementary file 11 [file elife-71888-fig5-video8.gif]

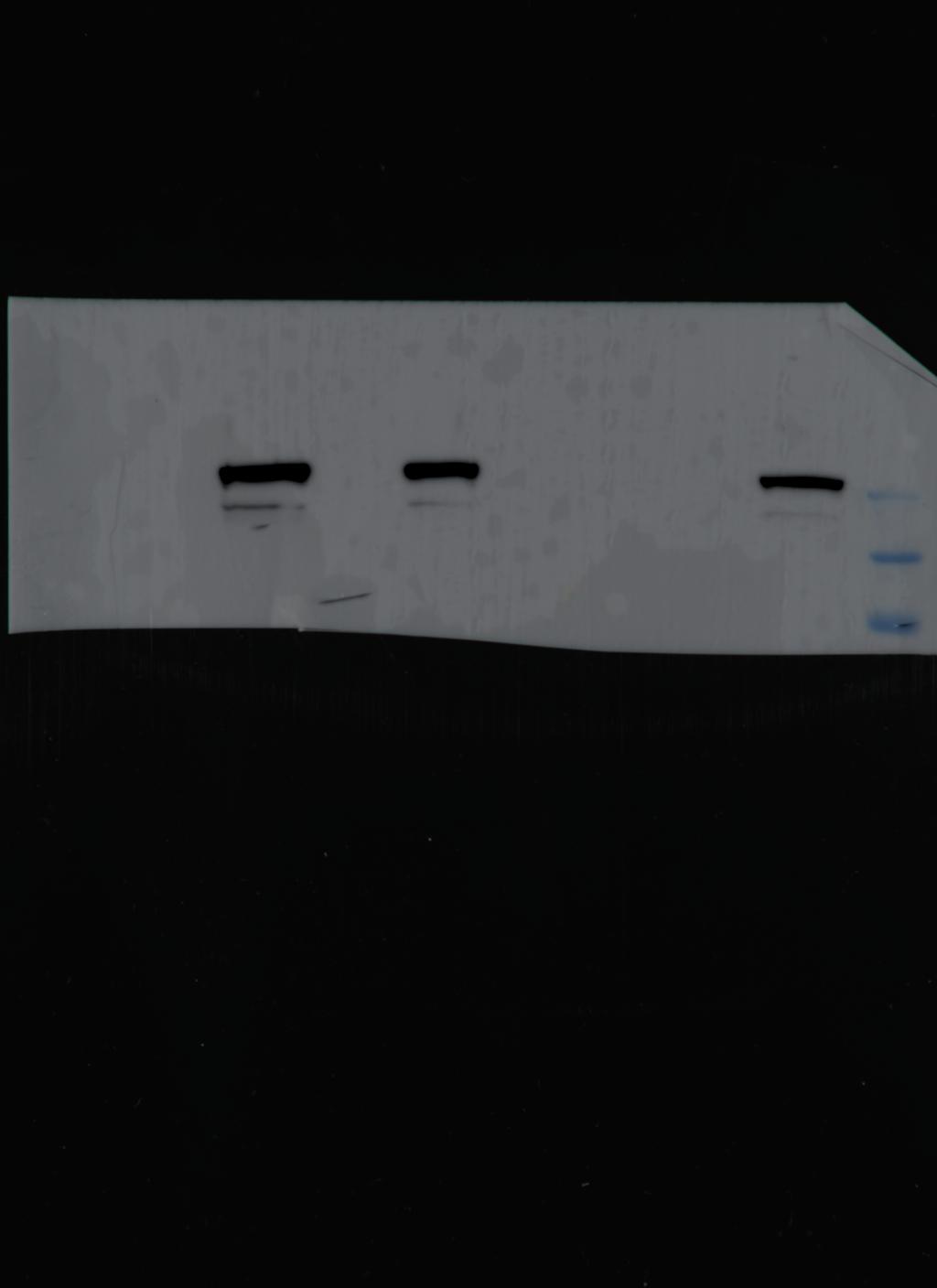

Supplement: Source data 2. [file elife-71888-data2.zip › Western Blots_raw images/Figure 1_figure supplement 1_NMHC IIA.jpg]

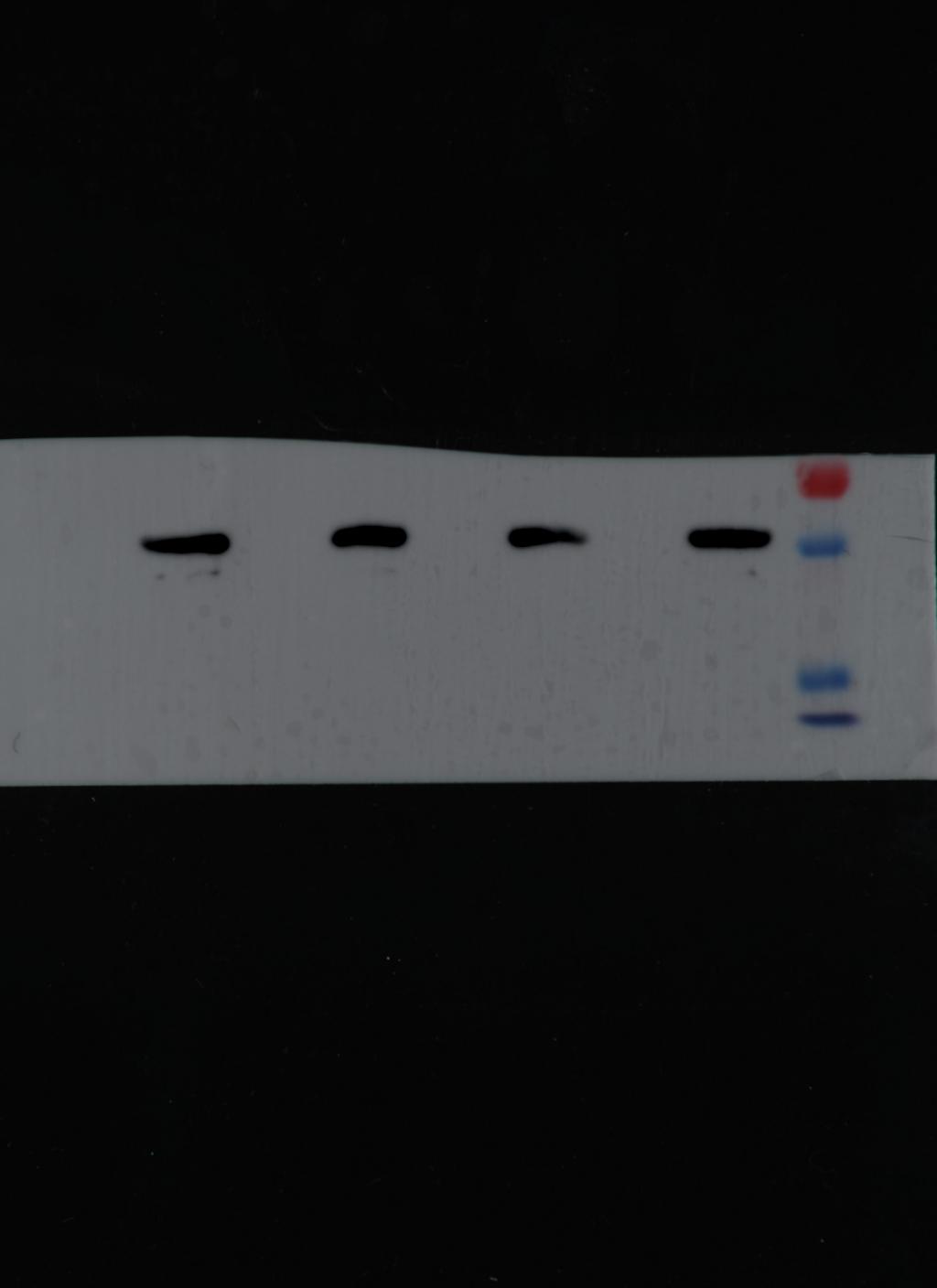

Supplement: Source data 2. [file elife-71888-data2.zip › Western Blots_raw images/Figure 1_figure supplement 1_NMHC IIA_Alpha-Tubulin loading control.jpg]

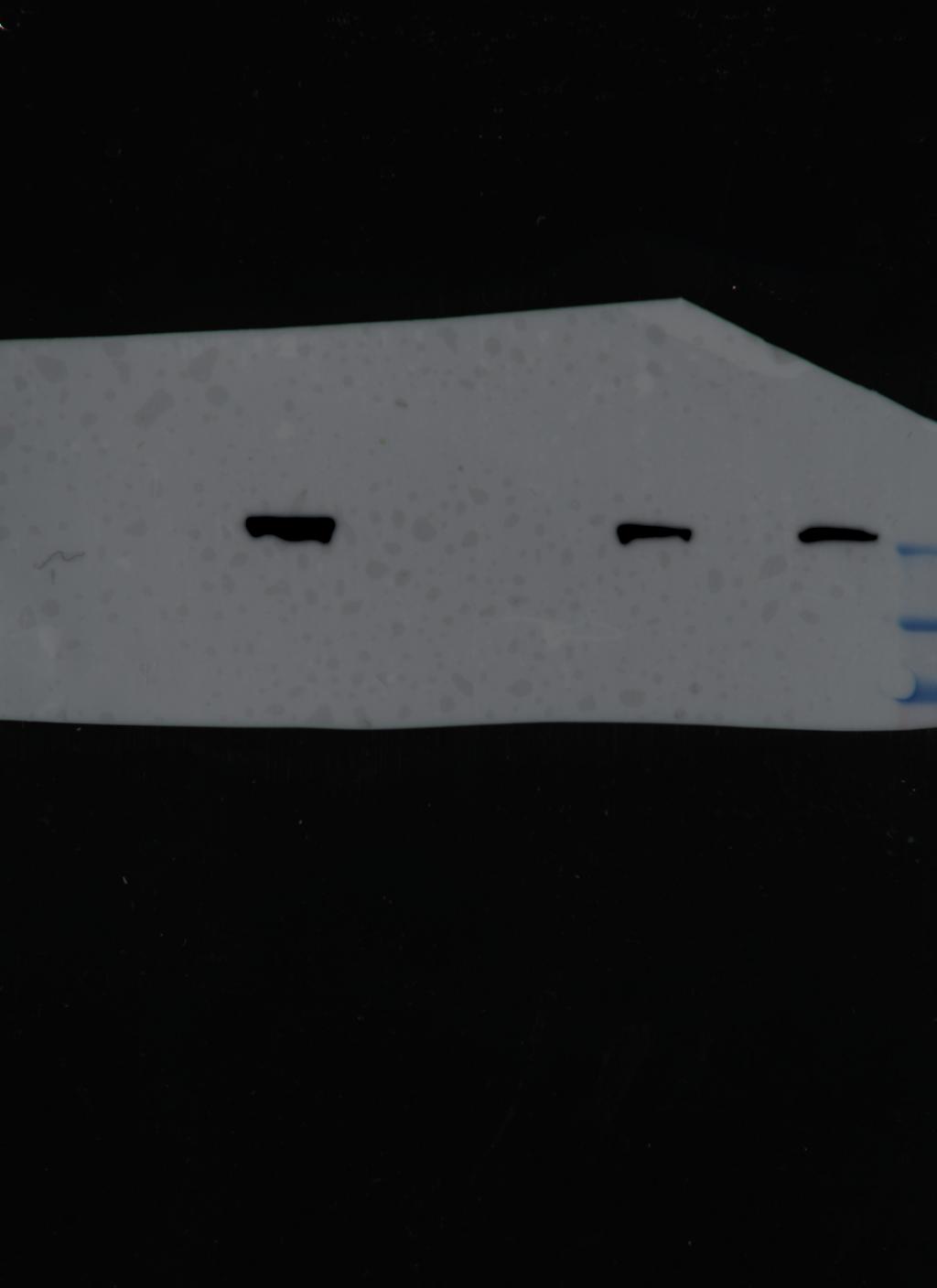

Supplement: Source data 2. [file elife-71888-data2.zip › Western Blots_raw images/Figure 1_figure supplement 1_NMHC IIB.jpg]

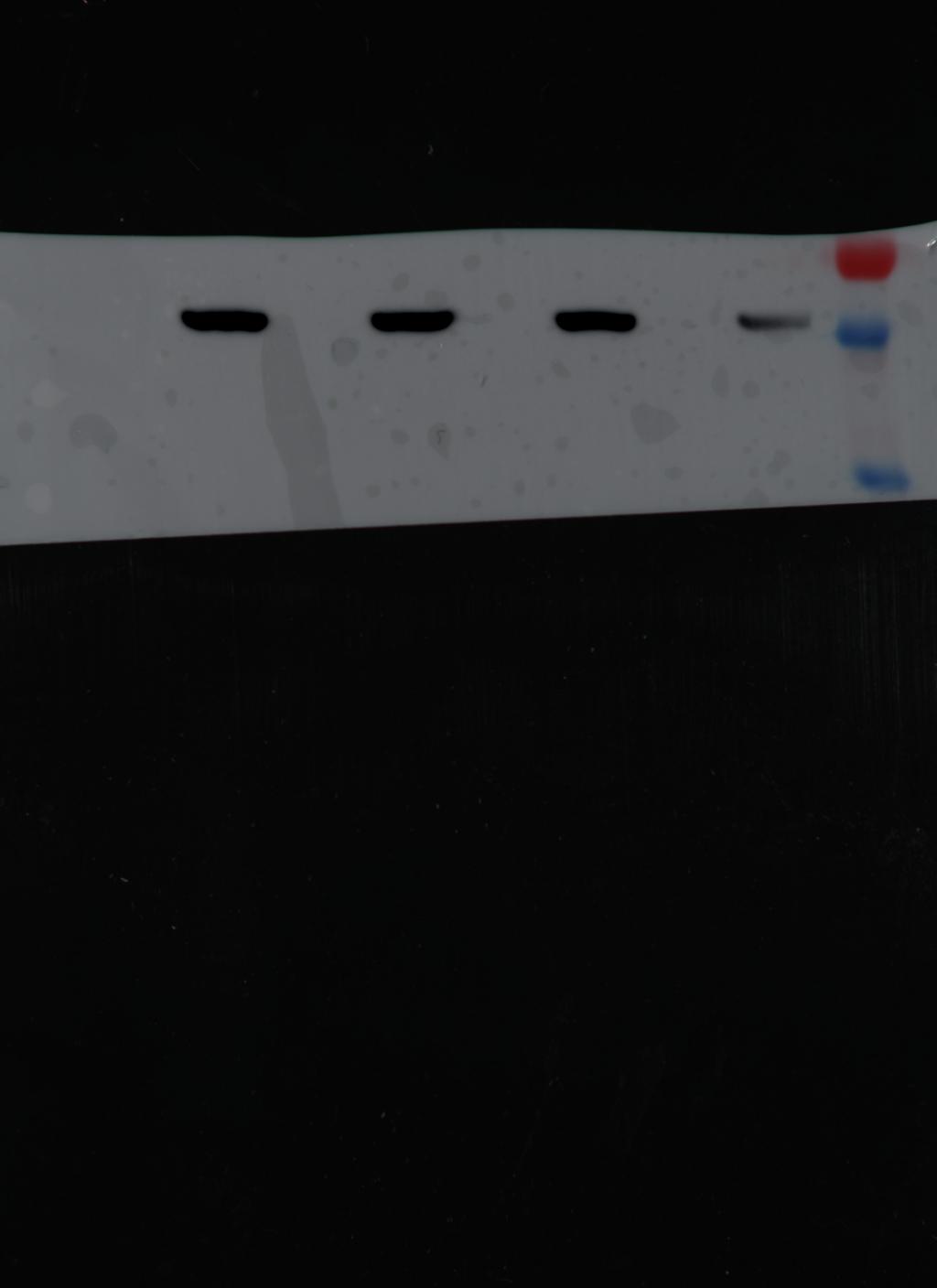

Supplement: Source data 2. [file elife-71888-data2.zip › Western Blots_raw images/Figure 1_figure supplement 1_NMHC IIB_Alpha-Tubulin loading control.jpg]

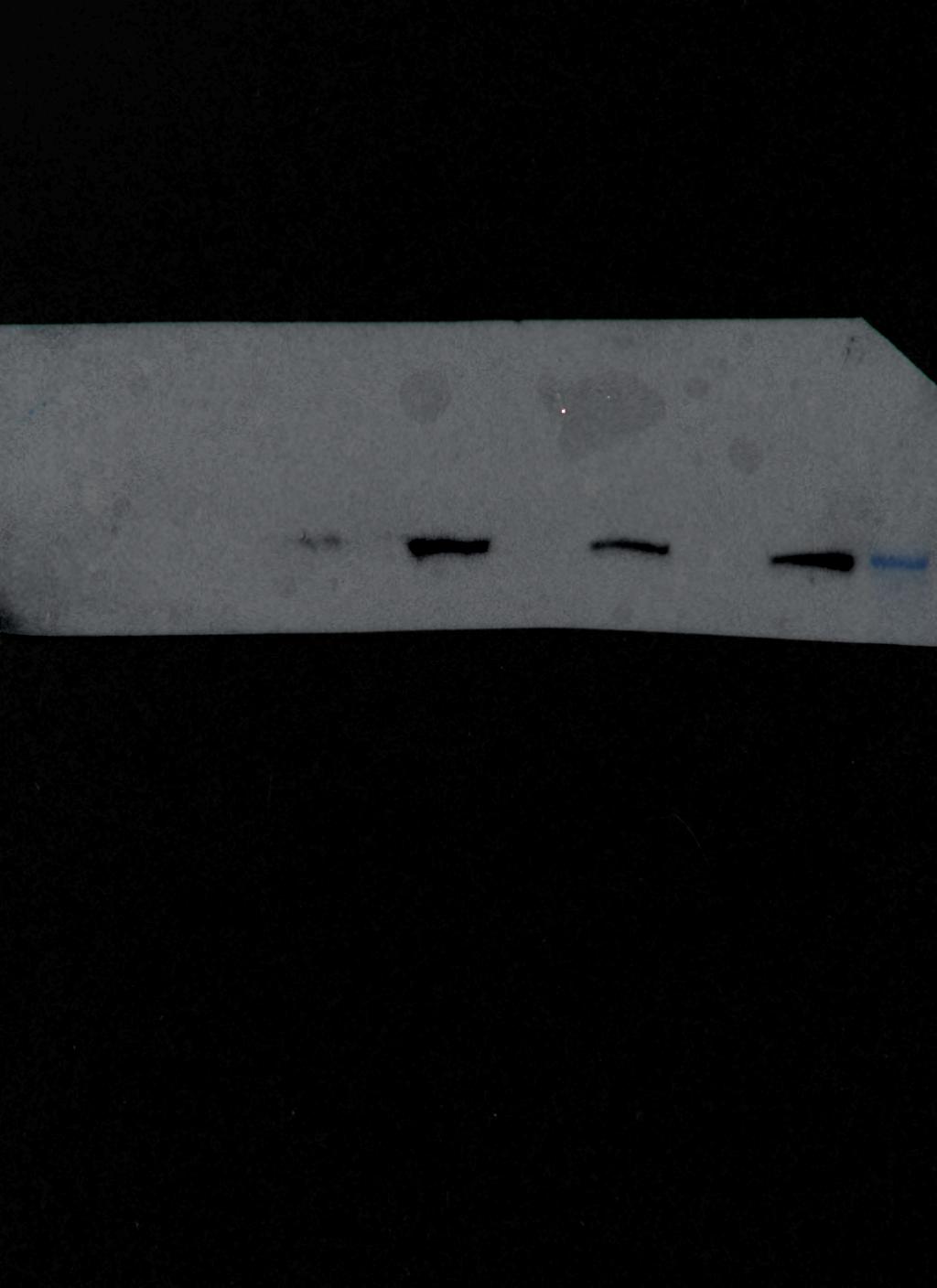

Supplement: Source data 2. [file elife-71888-data2.zip › Western Blots_raw images/Figure 1_figure supplement 1_NMHC IIC.jpg]

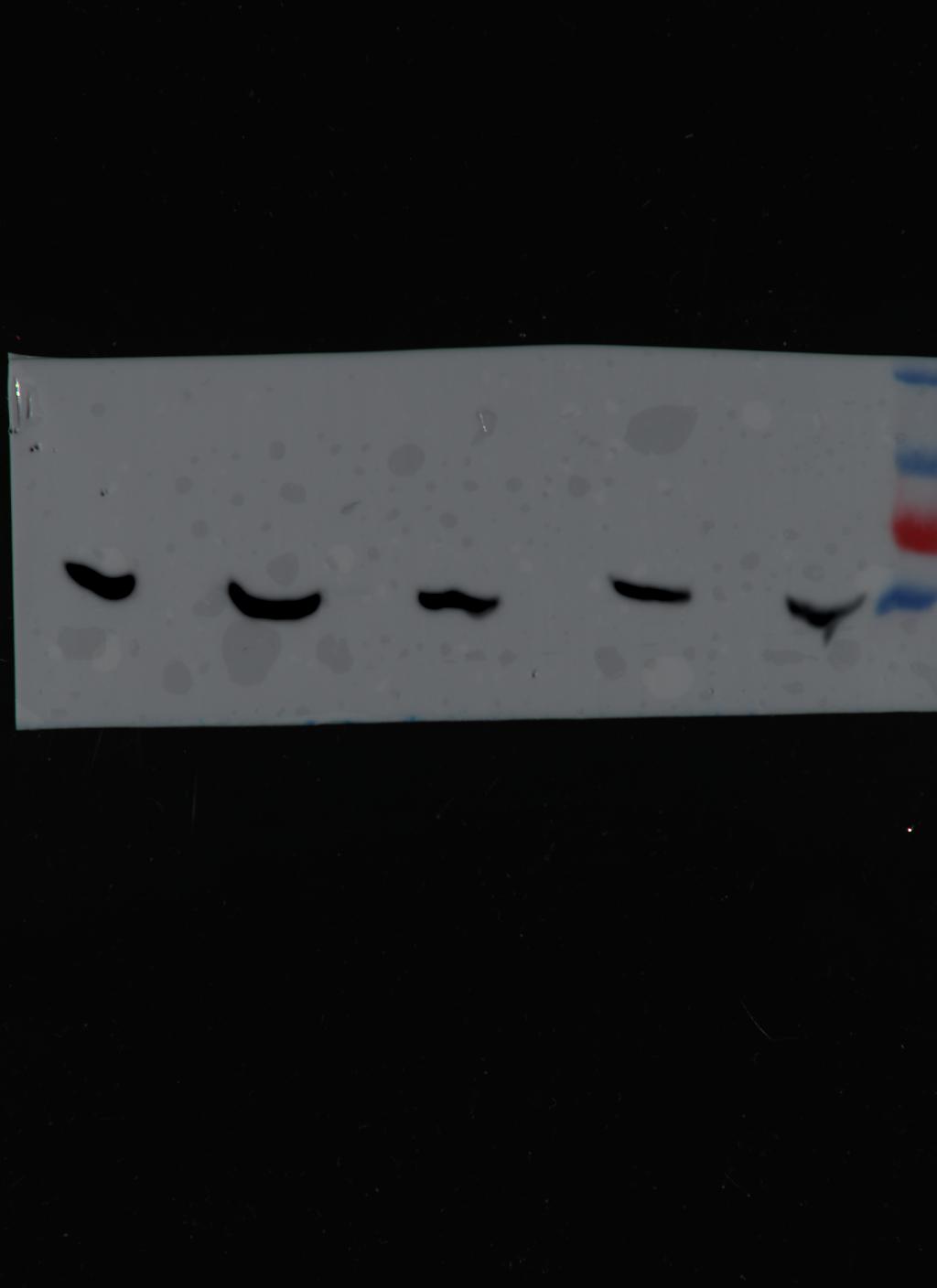

Supplement: Source data 2. [file elife-71888-data2.zip › Western Blots_raw images/Figure 1_figure supplement 1_NMHC IIC_Alpha-Tubulin loading control.jpg]

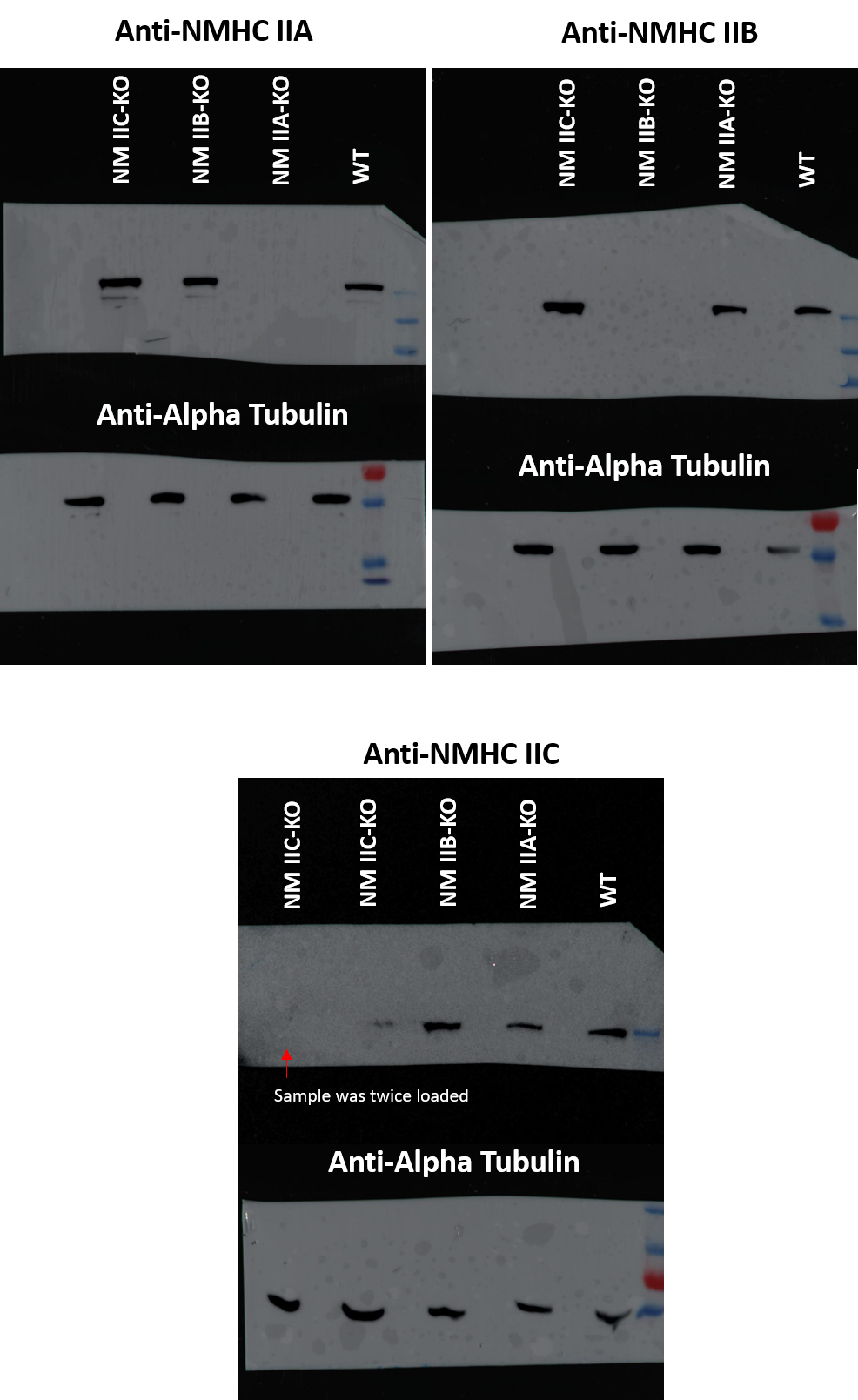

Supplement: Source data 2. [file elife-71888-data2.zip › Western Blots_raw images/Figure 1_figure supplement 1_Western Blot_full.tif]

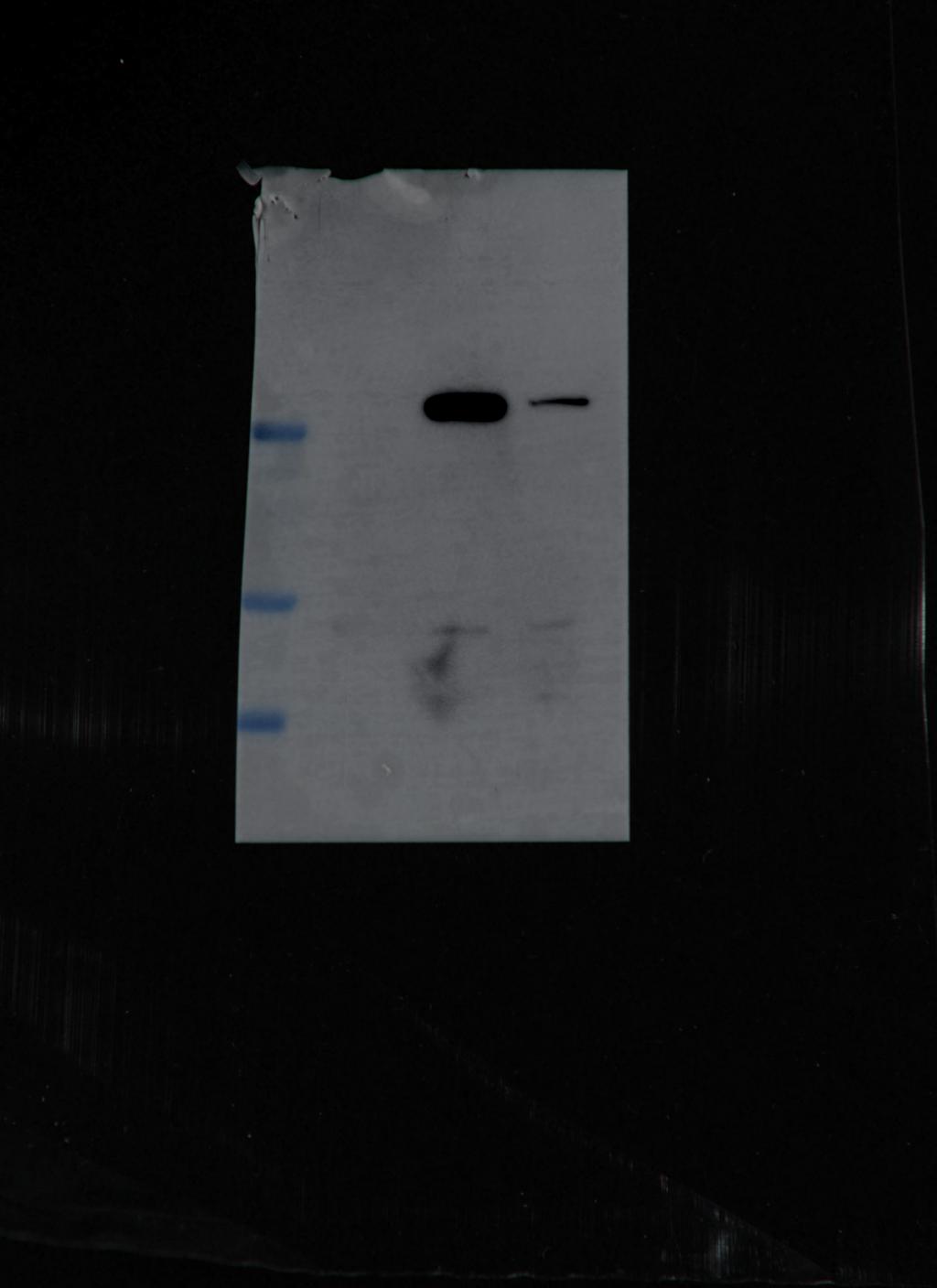

Supplement: Source data 2. [file elife-71888-data2.zip › Western Blots_raw images/Figure 2_figure supplement 1_GFP.jpg]

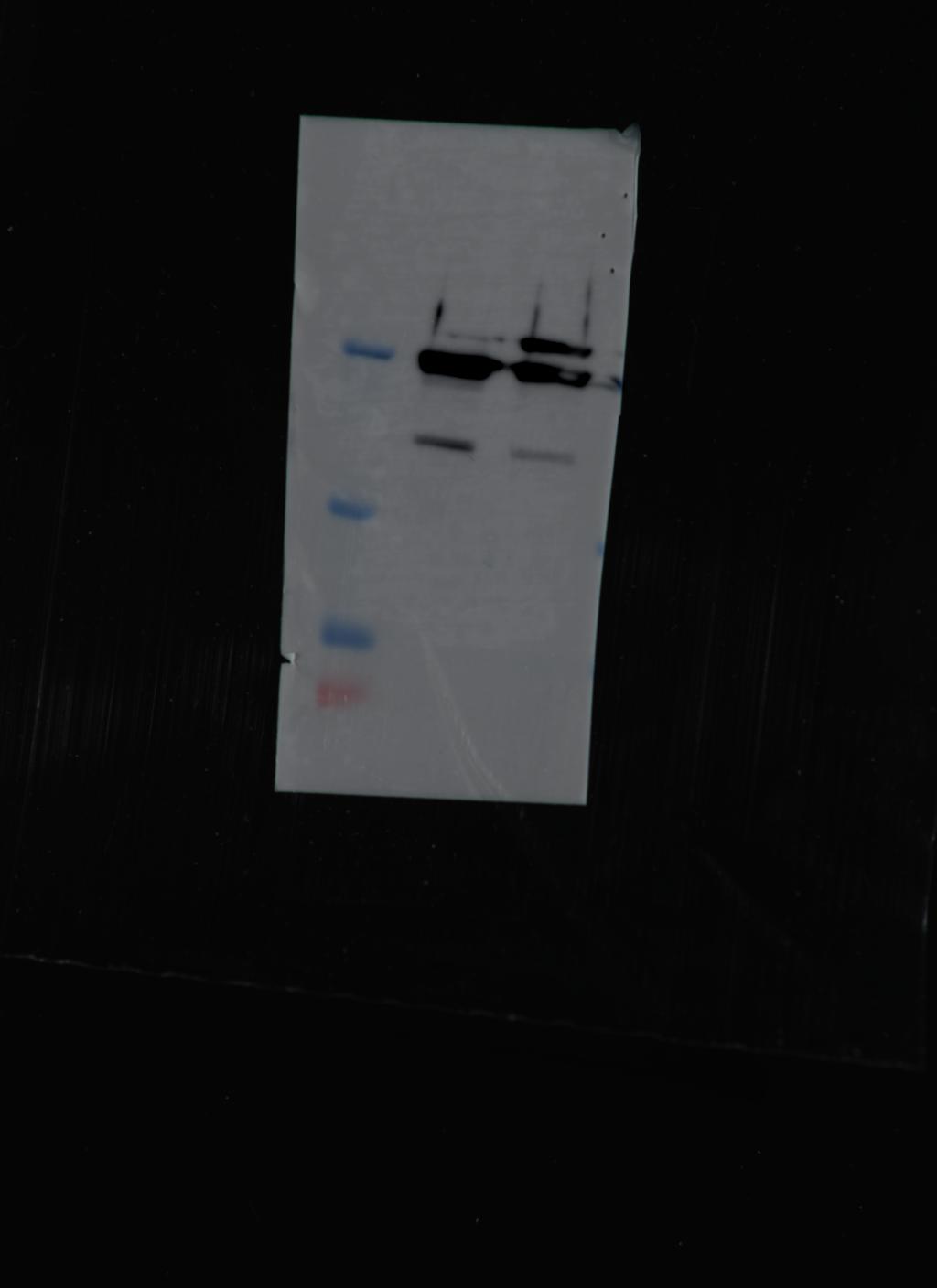

Supplement: Source data 2. [file elife-71888-data2.zip › Western Blots_raw images/Figure 2_figure supplement 1_NMHC IIA.jpg]

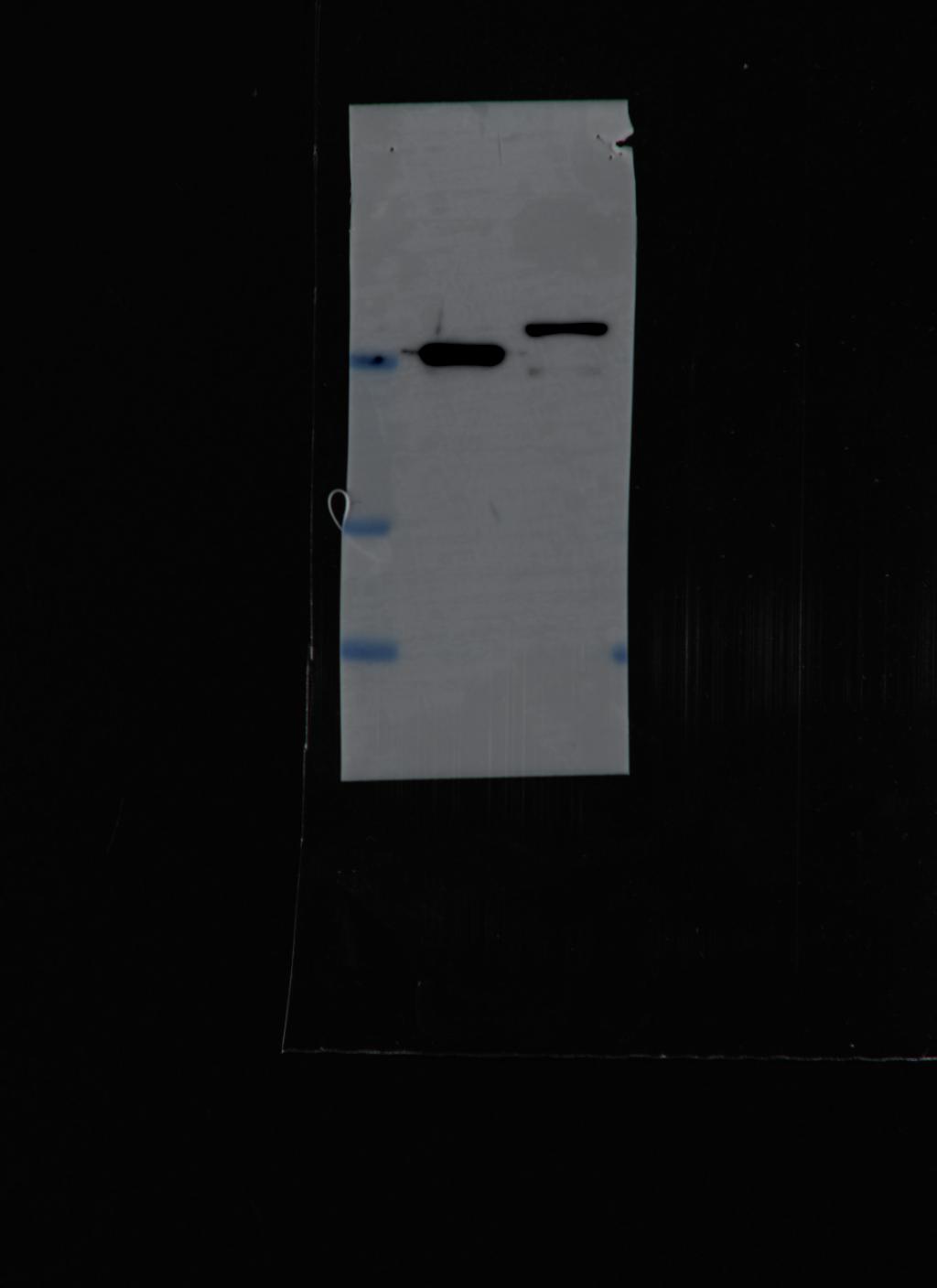

Supplement: Source data 2. [file elife-71888-data2.zip › Western Blots_raw images/Figure 2_figure supplement 1_NMHC IIB.jpg]

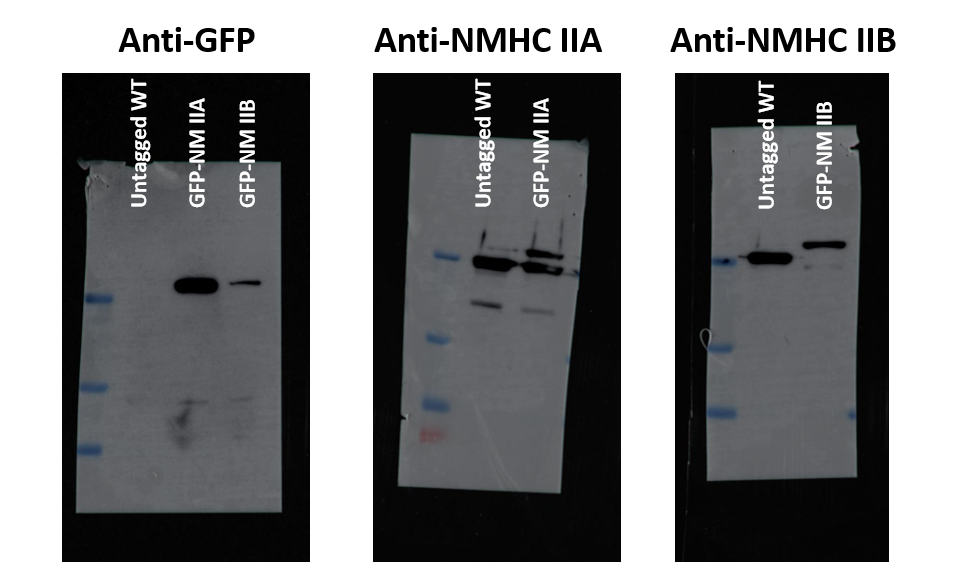

Supplement: Source data 2. [file elife-71888-data2.zip › Western Blots_raw images/Figure 2_figure supplement 1_Western Blot_full.tif]
