# Supplementary material for: Distinct roles of nonmuscle myosin II isoforms for establishing tension and elasticity during cell morphodynamics
Source: eLife. 2021 Aug 10;10:e71888. doi: 10.7554/eLife.71888 (PMC8391736; doi:10.7554/eLife.71888)
Supplement: Figure 5—source code 1. [file elife-71888-fig5-code1.zip › Source code/Digital Image Correlation/Correlation_Tracking_Guide_2010.htm]

Digital Image Correlation / Tracking with Matlab


Digital Image Correlation

and Tracking with
Matlab

 

 

 

 

 

 

 

 

 

 

 

 

 

 

 

 

 

 

 

 

 

Programmed by:

 

Chris Eberl, Robert Thompson, Daniel Gianola,
Sven Bundschuh

@ Karlsruhe Institute of Technology, Germany,
Group of Chris Eberl

@ Johns Hopkins University, USA, Group of Kevin
J. Hemker

 

chris.eberl@kit.edu or chris.eberl@jhu.edu

  

1. Introduction

 

Measuring strain in samples which are too
small, big, compliant, soft or hot are typical scenarios where non-contact
techniques are needed. A technique which can cover all that and also can deal
with complicated strain fields in structures or structural materials is the
Digital Image Correlation. With this technique, strain can be calculated from a
series of consecutive images with sub pixel resolution as will be shown in the
following chapters.

 

Even though there are tons of codes from the
image registration, artificial intelligence or the robotics community, none of
them can easily be used by the strain measuring community. Commercial code is
available also and has the advantage of getting a guaranty that it works, is
nicely designed and has well thought through user interfaces and typically a
higher processing speed. The disadvantages are, that commercial software
typically has to be paid in k$, is available only as package with hardware,
enjoys a notorious lack of programming interfaces or tools to change the code
to fit it into a test setup as well as the probability of inaccessible data in
case the software license is not valid anymore or it does not run on the new
and fancy computer anymore.

 

Out of all these reasons this code was started
together with Rob Thompson and Dan Gianola during my stay in the group of Kevin
J. Hemker at the Johns
Hopkins University
in Baltimore, MD, USA.
Considerable extensions and new code as well as all the multi core stuff was
added while Chris was heading a group (‘microreliability’)
at the KIT.

This code is not meant to be a direct
competitor to commercial code since we have not the time to make it as easy to
use as possible but as a different option with the advantages to be ‘free’,
‘flexible’ and ‘scalable’. ‘Free’ in terms of free access even though we would
like to ask you to cite our code in case you use it and ‘free’ again even
though you need to buy matlab together with some
toolboxes. Since most research institutions have access to this important tool
I think we still can name it ‘free’. ‘Flexible’ in terms of the relative easy
way you can enhance this matlab code as a script
language where you can add either other toolboxes or your own code to flex it
around your application. We would appreciate it if you as a user could share
your own code with all of us out here so we can learn from your creativity. And
‘scalable’ since you can easily start several sessions to process your images
on more than one processor (core) and the newest code segments are already
ready to use multicores and because you can also use
Graphic Processing Units (GPU = the graphics processor on a graphics card,
http://dside.dyndns.org/dict ) or other add-on boards to enhance processing
speed.

 

In case you are still reading, we would like to
wish you fun using this code and hope we were able to provide you with a useful
tool to help your with your experiments.

 

Cheers, Chris.

 

Karlsruhe, August 2010.

 

  

2. Requirements and
Installation

 

 

**REQUIEREMENTS:**

 

You will need **Matlab 7 (R14) or higher** (since the dlmwrite.m
in Matlab 6.5 does not work, at least for me and this is a really important
function since it stores the data after each calculation step). And you will
need the following **TOOLBOXES**:

 

-         
**Optimization** (all fitting processes depends on
this toolbox)

-         
**Image processing** (obviously)

-         
Optional
the **Parallel Computing Toolbox** (If
you want to use multicores)

 

You need to download the following .m files
from matlab central if you want a
full field interpolated strain field visualization:

‘surfit.m’, ‘polyfic.m’ and ‘polyvac.m’ from Vassili Pastushenko at the matlab central exchange into your matlab
work folder

 

 

**INSTALLATION STEP
1:**

 

Copy the files from the zip file you just
downloaded from the mathworks server into the work
folder in your matlab folder (e.g. in windows:
c:\matlab65\work or into your Matlab folder, in Windows it is found in your
Documents section):

 

-         
automate\_image.m                            (this
function does all the hard correlation work)

-         
automate\_image\_ld.m                       (is
used by large\_displ.m special version for large
displacement, needs automate\_image\_rf.m - this
function does all the hard correlation work)

-         
automate\_image\_mp\_2009b.m         (special version for multi processor
calculation - this function does all the hard correlation work)

-         
automate\_image\_rf.m                        (is
used by automate\_image\_ld.m)

-         
displacement.m                                  (this
function will help you analyzing your data)

-         
filelist\_generator.m                            (generates
file name lists with max. 8 letters and ‘.tif’ at the
end and creates a time\_image list needed for merging
stress and strain)

-         
gauss\_onepk.m                                  (the
gauss equation called by the peaktracking functions)

-         
gauss\_twopk.m                                  (same
as gauss\_onepk.m but with two peaks…)

-         
grid\_generator.m                               (generates
grid raster needed for the correlation code)

-         
jobskript.m                                         (script
to create batch process if you want to do a couple of folders)

-         
jobskript\_mp.m                                  (special
version for multi processor code - script to create batch process if you want
to do a couple of folders)

-         
large\_displ.m                                     (use
this if the displacement exceeds your correlation area)

-         
line\_visual.m                                      (needed
for the strain\_lineprofile.m)

-         
linearfit.m                                          (contains
the linear equation)

-         
local\_strainx.m                                  (this
function calculates the local resolved strain in the horizontal)

-         
Markerplotting.m                               (create
images with the position of the marker)

-         
multipeak\_tracking.m                        (track
multiple peaks along one axis)

-         
peak\_labelling.m                                (this
function is searching and tracking peaks)

-         
pickpeak.m                                        (similar
to peak\_labelling.m but you have to pick peaks by
yourself)

-         
ppselection\_func.m                           (this
function is needed by displacement.m)

-         
resume\_automate\_image.m               (restart
calculation if it has stopped by any reason)

-         
RTCorrCode.m                                  (“real-time”
correlation code)

-         
sortvalidpoints.m                               (this
function finds the tracked peaks and has to be called after peak\_labelling
or pickpeak)

-         
strain\_lineprofile\_marker.m               (tracking
two markers in a lineprofile)

-         
stress\_strainmatch.m                         (combines
correlation results and log file from tensile test machine)

-         
validxy\_mean.m                                (calculate
the mean over a couple of images)

**INSTALLATION STEP
2:**

 

In cpcorr.m (type
‘open cpcorr’ at the matlab
prompt) you have to change

 

-         
**in line 77:**

CORRSIZE = 5;

      to:

CORRSIZE = 15;

 

*(This changes the size of the selected parts of the image which will be correlated
from 10x10 pixels to 30x30. Change this to smaller values if you experience
slow computational speed or if you use low resolution images. Remember that
markers need more than double the space from its centre to the edge of the
image, otherwise they cannot be tracked.)*

 

-         
**in line 134 and 135:**

 

input\_fractional\_offset = xyinput(icp,:)
- round(xyinput(icp,:));

base\_fractional\_offset = xybase\_in(icp,:)
- round(xybase\_in(icp,:));   

      to:

input\_fractional\_offset = xyinput(icp,:)
- round(xyinput(icp,:)\*1000)/1000;

base\_fractional\_offset = xybase\_in(icp,:)
- round(xybase\_in(icp,:)\*1000)/1000;   

 

*(This is changing the resolution of the marker positions to 1/1000th
pixel. If you need higher resolution just increase these values)*

 

 

In findpeak.m
(which you will find in the private functions section off the Image processing
Toolbox folder).
The easiest way to get to it is to find it in cpcorr.m
in line 115, right click it and go to open selection. Sometimes you will need
to change the property settings so you can save it as a normal user. You can
also start Matlab as an administrator, change findpeak.m
and log in as a user again:

 

- line 58 and 59 from:

    x\_offset = round(10\*x\_offset)/10;

    y\_offset = round(10\*y\_offset)/10;   

 

to

    x\_offset = round(1000\*x\_offset)/1000;

    y\_offset = round(1000\*y\_offset)/1000;   

  

3. Good things to know about matlab:

 

The matlab help is
extremely helpful and should be the very first location you look in case of
errors. If you never worked with matlab but would
like to check it out, the ‘Getting Started’ is a good point to start with. I
started there in summer 2005.

 

**TAB:**

Pressing the TAB key on your keyboard after you
started typing in a command at the command line of matlab
will show you all functions with the same first letters.

 

**Arrow up function:**

Pressing the Arrow Up key on your keyboard
after you started typing a command will show the **last** command you started with the same first letters.

 

**Current Folder:**

The ‘Current Folder’ of matlab
is the folder on your harddisk which is currently
selected to process data in matlab (close to the
upper edge of the matlab window). Functions like ‘automate\_image.m’ require certain files to be present in
the ‘Current Folder’ otherwise they will produce an error (see description later
on). Pressing the little button on the right hand side with the three little
dots on it will let you select another folder. Another possibility is to use
the command window (see matlab help) or you can
select the current directory if it is activated under ‘View’ in that extra
window.

 

**Set semicolon:**

Set the semicolon after calling a function
(e.g. ‘automate\_image;’), otherwise all data which you get back from a called
function will be plotted in the command window.

 

**Workspace:**

The Workspace is the place where you can load
all your data into. Functions called by you will write their values into the
workspace and scripts will use the workspace all the time and leave a mess of
variables in there. If you do not know what is going on check out the ‘Getting
Started’ paragraph in the matlab help.

 

**How to load data?**

If you have loaded data into the workspace
(either by choosing ‘File’ à ‘Open…’ and selected the data file
you wanted to load or using the command window e.g. by typing: ‘load('filenamelist')’ and filenamelist
is present in the Current Directory) the data will appear in the workspace
window.

 

**How to saved data from
Workspace to the hard disk?**

If you want to save data from the workspace to
the hard disk, right click on it and select ‘Save Selection As…’. It will save the data with the matlab
file fomat. This data is only accessible by matlab. If you want to process the data also with other
programs you should consider to save the data as ASCII
file. Therefore type in the matlab console window ‘save('stress.txt','alltemp','-ASCII');’
to save the variable alltemp as text file  with the name ‘stress.txt’ to the ‘Current
directory’.  If you want to open it with matlab or excel you have to import the file. The delimiter
is per default TAB but can also be chosen to be comma or space (see matlab help).

 

**How to give data in
the workspace to the functions?**

If you type ‘displacement;’ into the command
window of matlab, the function will start and ask you
for the needed files. Instead you can load the data (e.g. validx.mat and
validy.mat) into the workspace and then give it to the displacement function by
typing: ‘displacement(validx,validy);’.
If have not loaded validx.mat there will be an error message.

 

**How do I get the data
from the function into the workspace?**

If you type ‘[validx,validy]=displacement;’ the variables validx and validy will be created
after running ‘displacement.m’. If you have
manipulated validx and validy
(e.g. if you cleaned up the data set from miss-tracked markers) you should save
them.

 

**Why are all images opened
in matlab mirrored to the horizontal center axis of
the image?**

Matlab reads in images like a diagram.
Therefore pixel (1,1) is in the lower left while image
processing software starts at the upper left corner.

 

**How to stop a function
in matlab?**

To stop a function press the control
key (‘Crtl’) together with ‘c’.

  

4. Digital Image
Correlation Quick Guide

 

This guide should help you to perform a simple
and fast analysis of your images. Before we start you should check your image
format and the naming of your files. The preferred image format is \*.tif and
can be compressed with the packbits compression. JPEG
or other image formats as well as MPEG video compression formats will not
provide you with sub pixel resolution since the images are processed to save as
much space as possible.

The script we use to create a list of images to
process (filelist\_generator.m) is kind of limited to
a certain format but it is possible to generate your own list of images. If you
want to change the format or the names of your images you can use free programs
like Irfanview (www.irfanview.com)
to batch process a huge number of images.

 

*4
Steps to Success:*

 

1.   
Step: Filename list generation with filelist\_generator.m

 

Just type ‘filelist\_generator;’
and press ‘ENTER’ at the command line of matlab. The
following window should appear:

 

 

If you select automatically, the
following menu will ask you for the first image you would like to process:

 

Here you can select e.g.
PIC0001.tif. You will be asked to save the file, please do
not change the name of the filename list as automate\_image
and other tools will need it. You should also save the file in the folder where
your images are. After that you will be asked if you want to read out the time
from the images:

 

 

If you answer yes Matlab can read
the acquisition time from your images. As the information is gathered fro the EXIF data the time resolution is limited to 1
second.

 

If you chose to use manual you will
find a different window:

 

 

Fig. 1: Input of first and last image to create
an image list with filelist\_generator.m

 

The numbers will be the number at
the end of each filename. After depositing these numbers in the dialog the next
window will ask for the first 4 letters of the filenames.

 

 

Fig. 2: Input for the first 4 letters in filelist\_generator.m

 

The next step is to save the file
name list into the folder with the images to process.

 

Fig. 3: Dialog to save the file name list into
the folder with the images to analyze.

 

 

 

1.   
A) Grid generation with grid\_generator.m
for correlation (if you expect large displacement go directly to B!):

 

It has to be noted that the user can
always generate his own marker positions. Therefore the marker position in pixel
has to be saved as a text based format where the x-position is saved as
‘grid\_x.dat’ and the y-position saved as ‘grid\_y.dat’.

To start just type ‘grid\_generator;’ and press ‘ENTER’ at the command line of matlab. The following window should appear:

 

 

Fig. 4: Dialog to open the first (base) image
to generate a grid

 

In this dialog the first (base)
image can be selected in which the grid can be created. After selecting this
base image, the image will be opened and a new dialog pops up to ask you if you
would like to load an existing grid. If you want to create a new one just hit
No and go ahead.

 

 

 

Fig. 5: The opened base image and the menu to
select a preexisting grid.

 

You will get a new choice where you
can select a shape for your grid.

 

 

The different types are a
rectangular or circular grid, two markers, or a line of markers. If you choose
a rectangular grid type, the pointer will change from an arrow to a horizontal
and a vertical line which will help you finding the right position. The idea is
to click on the two diagonal positions which will define the outer dimensions
of a box containing the grid.

 

 

Fig. 6: The horizontal and vertical lines allow
an accurate positioning of the grid.

 

The selected box will be shown in
the image and a dialog will pop up to ask your for the input of a raster point
distance in x and y direction.

 

 

Fig. 7: Horizontal (x-direction) and vertical
(y-direction) grid resolution with a default resolution of 50 pixel distance
between raster points.

 

The code will now generate the chosen
grid and will plot it on top of the sample image. The last dialogue will ask
you if you want to use the generated grid and save the grid\_x.dat and
grid\_y.dat to be processed, if you want to try again or if you want to choose
another grid type.

 

 

Fig. 8: The last menu will allow you to accept
the grid (which will be saved), try again or choose another grid type.

 

You will be asked if you would like
to add more points ro if you would like to remove
markers. If you are happy with the result just hit END.

 

 

B) Calculate
Correlation with large\_displ.m:

Large\_displ.m was written to compensate for large
displacements between images. This can be due to a low image acquisition rate
or large oscillations. Therefore we do shrink the images and let the code
determine the displacement of the images relative to each other and let this
feed back into the second round. There the code continues as described in the
normal automate\_image.m script with a dense grid. You
need to provide two different grids. Large\_displ.m
will guide you through the steps, first you will be asked for a reduction
factor and a coarse grid (saved in grid\_x\_small.dat and grid\_y\_small.dat) for a
pre-calculation of the displacement in the images. Then you will be asked for a
fine grid to calculate the displacement in the original images.

 

Fig. 9: Enter the reduction factor to shrink
the original images, e.g. a factor of 5.

 

Fig. 10: Press “OK”

 

Fig. 11: Select your first image.

 

In this dialog the first (base)
image can be selected in which the grid can be created. After selecting this
base image, the image will be opened and a new dialog pops up to ask you if you
would like to load an existing grid. If you want to create a new one just hit
No and go ahead.

 

 

Fig. 12: The opened base image and the menu to
select a preexisting grid.

 

You will get a new choice where you
can select a shape for your grid.

 

Fig. 13: Create a coarse grid for a
pre-calculation.

 

The different types are a rectangular
or circular grid, two markers, or a line of markers. If you choose a
rectangular grid type, the pointer will change from an arrow to a horizontal
and a vertical line which will help you finding the right position. The idea is
to click on the two diagonal positions which will define the outer dimensions
of a box containing the grid.

 

Fig. 14: The horizontal and vertical lines
allow an accurate positioning of the grid.

 

The selected box will be shown in
the image and a dialog will pop up to ask your for the input of a raster point
distance in x and y direction.

 

 

Fig. 15: Horizontal (x-direction) and vertical
(y-direction) grid resolution with a default resolution of 50 pixel distance
between raster points. As mentioned before use 50-100 markers
for the whole image.

 

The code will now generate the chosen
grid and will plot it on top of the sample image. The last dialogue will ask
you if you want to use the generated grid and save the grid\_x\_small.dat and
grid\_y\_small.dat to be processed, if you want to try again or if you want to
choose another grid type.

 

 

Fig. 16: This menu will allow you to accept the
grid (which will be saved), try again or choose another grid type.

 

You will be asked if you would like
to add more points or if you would like to remove markers. If you are happy
with the result just hit END.

 

Fig. 17: Main menu of the grid generator.

 

All this work was done for the
pre-calculation of the large displacement, now do the same for the accurate
analyze of the original images.

 

 

Fig. 18: Press “OK”. Then select the first
image for your analysis.

 

Fig. 19: Select your first image, same as for
the coarse analysis.

 

Fig. 20: If you want to use an old grid press
“Yes” and select your old gridx.dat and gridy.dat files otherwise press “No”.

 

Fig. 21: Create a grid for the image
correlation calculation. Here you can use as many markers you want, if you have
enough time ☺. Finish with “END”.

 

The Correlation calculation will
start immediately. Go on with point 4.

 

2.   
Run correlation with automate\_image.m
or with automate\_image\_mp\_2009b.m:

 

The automation function is the central
function and processes all markers and images. Therefore the ‘Current
directory’ in matlab has to be the folder where automate\_image.m finds the filenamelist.mat, grid\_x.dat and
grid\_y.dat as well as the images specified in ‘filenamelist.mat’. Just type ‘automate\_image;’ and press ‘ENTER’ at the command line of matlab.

At first, automate\_image.m
will open the first image in the filenamelist.mat and plot the grid as green
crosses on top. The next step will need some time since all markers in that image
have to be processed for the first image. After correlating image one and two
the new raster positions will be plotted as red crosses. On top of the image
and the green crosses. The next dialog will ask you if you want to continue
with this correlation or cancel. If you press continue, ‘automate\_image.m’
will process all images in the ‘filenamelist.mat’. The time it will take to
process all images will be plotted on the figure but can easily be estimated by
knowing the raster point processing speed (see processing speed).

Depending on the number of images
and markers you are tracking, this process can take between seconds and days.
For 100 images and 200 markers a decent computer should need 200 seconds. To
get a better resolution you can always run jobs overnight (e.g. 6000 markers in
1000 images) with higher resolutions.

Keep in mind that ‘CORRSIZE’ which
you changed in ‘cpcorr.m’ will limit your resolution.
If you chose to use the 15 pixel as suggested a marker distance of 30 pixel will lead to a full cover of the strain field.
Choosing smaller marker distances will lead to an interpolation since two
neighboring markers share pixels. Nevertheless a higher marker density can
reduce the noise of the strain field.

When all images are processed, automate\_image will write the files validx.mat, validy.mat,
validx.txt and validy.txt. The text files are meant to store the result in a
format which can be accessed by other programs also in the future.

 

To stop automate\_image
use the key combination ‘*Ctrl* c’.

In case of a crashes, errors
generating the right filenamelist or the interruption
of the correlation process by the user, the function ‘recover\_correlation.m’
can be called which extracts the last marker positions sets up ‘automate\_image.m’ and continues at the last image.

 

3.   
Analyze the displacement with displacement.m:

 

As last part, the post processing is
the most interesting and awarding step since you actually can analyze the
collected displacement data. The displacement.m
function is a small collection of functions which allows you to review the
displacement field, calculate the strain or delete markers which were not
correlated or tracked very well.

To start, type into the command
window ‘displacement;’ or ‘[validx,validy]=displacement;’
in case you want to save the changed files (validx
and validy) from the workspace (see chapter 3). A
window will pop up asking you for the validx.dat file which contains the
x-displacement off all markers in all images, followed by a dialog for the
validy.dat containing the y data. After ‘displacement.m’
has loaded both files, a new window pops up which allows you
to choose between different options.

 

 

Fig. 22: The displacement.m
function allows cleaning up the data set selecting parts of it plot the
displacement or measure the strain in x- and y-direction.

 

For a typical analysis you always
have to delete some of the markers with did not all too well during the
correlation or peak fitting step. This can happen e.g. due to marker movement
during the test, changing light conditions or in case of the correlation
technique due to the fact that the sample surface did not provide enough
characteristics.

We start with clicking on the **‘3D Mesh Plot of Displacement’** button
which will bring up a new window and a dialog asking if you want to create a
video. Clicking on ‘yes’ will create a new folder called video and the 3D
displacement plot of each image will be saved as \*.jpg. The click on the no
button will just start the 3D displacement plot. This part of the ‘displacement.m’ allows you to watch displacement (z-axis)
versus location (x- and y-axis) for all images. To get a better 3-dimensional
understanding all markers are projected as green dots on the plane normal to
the y axis. The Image number will be shown in the plot. It has to be noted that
the orientation of the strain depends on the orientation of the image during
the correlation process. The x-axis in the plot is the horizontal direction in
the image and the y-direction the perpendicular direction. The plotted
displacement on the z-axis is always the x-displacement of the data contained
in validx.mat and validy.mat. To look at the y displacement the user has to
wait for all images to be plotted and then after the displacement-dialog
appears again, click the button ‘**Rotate
Orientation (exchange x and y)**’. This will exchange validx
and validy and clicking again on the **‘3D Mesh Plot of Displacement’** button
will now show the displacement in the y-direction. The user has to keep track
of this change since it will affect all plotting and strain measurement steps
lying ahead during the same ‘displacement.m’ session.

 

 

Fig. 23: 3D Displacement versus x- and
y-position. The orientation of the x-axis is the horizontal in the analyzed
image and the y-axis is the vertical. The displacement is always the x-diplacement until you exchange validx
and validy with the ‘**Rotate Orientation (exchange x and y)**’ button.

 

The next step is to get rid of badly
tracked markers. This can be done in three different ways:

-         
delete
single markers, click ‘**Remove badly
tracked marker, one by one (Position)**’

-         
delete
a bunch of markers at once, click ‘**Delete
multiple markers (Position)**’

-         
delete a bunch of markers from the displacement
versus x position plot, click ’**Delete
markers from displacement vs. position plot**’.

The first two will provide you with
a top down view with x- and y axis being the horizontal and vertical direction
in the analyzed image and the displacement is expressed as underlying colors.
The third option will show the projected markers which allows
you to sometimes more easily access the peaks. You have to play around with
these two different views and rotate the orientation back and forth while you
are checking through the images until you get a clean data set. This requires
some practice, therefore take your time and analyze the data carefully before
you really delete a bunch of markers.

‘**Remove badly tracked marker, one by one (Position)**’ will allow you
to click on markers which are not at the right place. The marker with the
highest displacement value will be a red dot while the marker with the lowest
displacement will be a blue dot. By clicking close to one of the markers the
plot will be updated to the new displacement field and the color code will be
updated to the new highest and lowest displacement value.

‘**Delete multiple markers (Position)**’ will allow you to choose a
rectangle and all markers in it will be deleted.

The same applies to the ’**Delete markers from displacement vs.
position plot’,**
it is just a different view of the markers.

**Delete markers moving relative to their neighbors** which finds the next 10 or 20
neighbor markers and plots the relative distance to these guys. If you acquired
a lot of markers and images that may take a while. If you want a noise free
image this tool allows you to find the bad guys jumping around between
positions:

The red line markes
the maximum allowed difference to the other markers and the cross is where your
mouse is hovering. If you click a second time where the mouse is in the figure,
the minimum and maximum allowed relative displacement to the next neighbors
would be just 0.11 pixels. This function helps a lot cleaning up your data set.

 

After cleaning up the data set and
if you have started the ‘displacement.m’ file with ‘[validx,validy]=displacement;’, you
will find validx and validy
as variables in the workspace. Right-click on them and save the selection with
a different name than validx.mat and validy.mat and nect
time you open up ‘displacement.m’ choose them.

 

After cleaning up and saving the
data you will want to measure strain. Here also two ways you can choose. The
first one is straight forward by just clicking on either ‘**Strain Measurement between 2 Points**’ which will let you choose two
points or ‘**1D Average Strain Measurement**’
which will use all points available. The second one is to select markers with ‘**Select Markers to Analyze**’ from a
certain location and then jump back to the ‘displacement.m’
and then calculate the strain. Again it is important to keep track of all ‘**Rotate Orientation**’ operations since
you will analyze in both cases the x-displacement versus the x-position. In
case the data was not rotated, the strain in horizontal direction in the image
will be measured.

 

After clicking on
‘**Strain Measurement between 2 Points**’
you will have to choose two points. The function will find the closest two points
to where you clicked and plot the strain versus image number.

If you are not happy with the result
you can still choose two other markers by clicking ‘Yes’ in the next dialog. If
you stick with these markers, you can either save the result as a text file
(‘image\_1Dstrain.txt‘) or just go back to the ‘displacement.m’
dialog. Make sure you change the filename in the explorer before you run this
code another time since it will just overwrite it.

 

 

 

Fig. 24: From this window two points can be
picked which will be used to measure the strain

 

 

Fig. 25: Strain versus image number

 

After clicking on the ‘**1D Average Strain Measurement**’ button,
the x-displacement versus x-direction will be plotted for each image and then
fitted by a linear function. The slope is the true strain which will be plotted
versus the image number after all images are processed. If you choose to save
the strain versus image number you will be asked where you want to save the
data as an ASCII file which can be opened with matlab,
excel or just the notepad.

 

 

 

Fig. 26: The slope of the linear fit of the
displacement versus position allows to plot the true
strain versus image number.

 

 

If you want to analyze a special
part of your sample it is best to use the ‘**Select
Markers to Analyze**’ button in the ‘displacement.m’
menu. The menu which will pop up allows you to choose between different types
of grids.

After you have chosen the markers
you want to process, run ‘**1D Average
Strain Measurement**’ to get the strain from these markers. Clicking the ‘**Rotate Orientation (exchange x and y)**’
button and running the strain analysis again will give you the strain in the
perpendicular direction.

  

5. Digital Image Tracking
Quick Guide

 

This guide should help you to perform a simple
and fast analysis of your images. Before we start you should check your image
format and the naming of your files. The preferred image format is \*.tif and
can be compressed with the packbits compression. JPEG
or other image formats as well as MPEG video compression formats will not
provide you with sub pixel resolution since the images are processed to save as
much space as possible. The script we use to create a list of images to process
(filelist\_generator.m) is kind of limited to a
certain format but it is possible to generate your own list of images which
will be explained later. If you want to change the format or the names of your
images you can use free programs like Irfanview (www.irfanview.com) to batch process a huge
number of images.

 

*4
Steps to Success:*

 

1. Step:
Filename list generation with filelist\_generator.m

 

Just type ‘filelist\_generator;’
and press ‘ENTER’ at the command line of matlab. The
following window should appear:

 

 

Fig. 1: Input of first and last image to create
an image list with filelist\_generator.m

 

The numbers will be the number at
the end of each filename. After depositing these numbers in the dialog the next
window will ask for the first 4 letters of the filenames.

 

 

Fig. 2: Input for the first 4 letters in filelist\_generator.m

 

The next step is to save the file
name list into the folder with the images to process.

 

Fig. 3: Dialog to save the file name list into
the folder with the images to analyze.

 

 

2.   
Run tracking with peak\_labelling.m,
pickpeak.m and strain\_lineprofile.m:

 

**‘peak\_labelling.m’**

If you trust the automatic peak
labeling, you can use ‘peak\_labelling.m’. Type ‘[validx,validy]=peaklabelling;’
It will scan your image and subtract the hopefully dark background and identify
maxima with a value higher then a certain grey value.
It will ask you for an image (the base image) which will be used to identify
the peaks and run a first fit through all of them.

 

 

Fig. : Open the first image for
automatic peak identification.

 

After opening the first image ‘peak\_labelling’ will plot the image as intensity plot where
blue is low and red is high intensity. You will be asked to draw a box in which
‘peak\_labelling’ will check for peaks. After
selecting the area, it will take some time to process. After identifying the
peaks ‘peak labeling will automatically start to fit all peaks and plot the
residuals of all peaks. Minimizing the matlab console
window will increase processing speed. The title in the figure will indicate
the status of the processing and the estimated time it will take.

 

 

Fig. : Select an area to find peaks.

 

At the end of the processing, all
relevant files (‘fitxy.dat’ contains the fitting parameters for each point,
‘validx.dat/.mat’ contains the x-position of each peak, and ‘validy.dat/.mat’
contains the y-position of each peak) will be saved in the current folder. One
relevant parameter is not directly accessible since this approach is trying to
automate the whole processing step but can be changed in the ‘peak\_labelling.m’ file. 
It can be found in ‘peak\_labelling.m’ in line
182 and 183 where the residuals of the fits in x and y directions are validated
to guarantee a flawless processing. If a fit does not work at all, the function
will crash. To prevent this I let the function decide very early which peak is
good or bad. Only the fits with a low residual will be used. Therefore you
should make sure you do not use too high values here. You will also find a
similar value in the function ‘sortvalidpoints.m’
which is called by ‘peak\_labelling.m’ at the end of
the processing to create ‘validx’ and ‘validy’. You have to change this value in line 39 too, otherwise the peaks will be deleted at the end. Since
the input file fitxy.dat is saved before these points are deleted, you can
still play around with this value and see how it affects your resulting validx and validy.

 

 

**‘pickpeak.m’**

After starting the function by
typing at the console ‘pickpeak;’,
the function will ask you for the first image and then will need to know how
many peaks you want to identify.

 

 

Fig. : How many peaks do you want to identify for
tracking by ‘pickpeak.m’?

 

After you typed in a number (e.g.
20), the function will present you the selected image and you can click boxes
around the peaks you want to track. For each peak you have to define a box by
clicking on the lower left and the upper right of each peak. The center of each
box will be highlighted by a blue circle. It is very important that you choose
a box which is wide enough for the curve fitting to get enough data points. But
if you choose too big boxes you will trap several peaks in them and the
residual of the fit will be high which the software will interpret as bad fit.
A box size of 2-4 times of the visible peaks seems to be a good idea. Also it
is better to choose round shaped peaks since this provides a better greyscale profile if you choose to use a gauss function for
the fitting process.

After you picked all peaks, the
software will fit all peaks which will be displayed in a small window and after
the first image processed you will only see the actual image and with blue
circles on top indicating the peaks which are still in the fitting process.
Vanishing circles indicate that the peak could not be fitted any more.  The title in this window will tell you the
approximated total processing time and how much percent of the images are
processed.

After all images are processed, the
data will be saved the same way as in the ‘peak\_labelling.m’
function.

 

**‘strain\_lineprofile.m’**

This function will track two greyscale maxima in a line profile which you can choose
from an image. After opening the first image, the software will let you to
choose a horizontal line at a vertical position in the image. The next dialog
will ask you which integration width (in vertical, y-direct) you want to use.
Default is 40 but you should keep in mind that it should be either much wider
or much narrower than your markers. If you choose the same width and the
markers are drifting in y direction the peaks in the greyscale
profile will change which will translate as error into your strain analysis.

The calculated greyscale
profile will then be plotted and you can choose two peaks. The first click
should be located on the horizontal level of the background and the vertical
position of the first peak and the second click should be placed at the
horizontal level of the average peak amplitude of the two chosen peaks and the
vertical position of the second peak. After the second click, the function will
fit two gauss functions to the greyscale profile and
plot a red fitting function on top of the data while processing all images. The
peak positions will be saved in the file ‘raw\_peak\_results.dat’ and the strain
as strain\_x.dat as well as a two column file with the image number in the first
column and the strain in the second column. All files are tab delimited ASCII
format and can be opened e.g. with excel. You cannot use ‘displacement.m’
for the strain analysis since this data is only 1D with 2 points.

 

Fig. : Greyscale
lineprofile, ready to pick two peaks.

 

3.   
Run displacement.m:

 

Please check step 4 in ‘4. Digital
Image Correlation Quick Guide’.

  

6. Extra scripts and
information you might find useful

 

 

**‘stress\_strainmatch.m’**:

Matching stress and strain can become a pain if
they were captured with different programs and/or computers, which can be the
case if the strain is captured with a camera. This little script can read in
stress and strain files (as long as they are ASCII files) and match the two
together. It needs the ‘time\_image.txt’ which is created by the ‘filelist\_generator.m’, the strain file and the stress file.
You have to choose which column is stress and strain in each file. After it has
loaded all the files the script will ask you for the time between starting the
stress measurement and the first image file. The stress versus image plot shows
you immediately if the chosen value makes sense and the file
‘stress\_image\_x.txt’ will be written to the ‘Current Folder’ on the hard disk.

 

**‘Markerplotting.m’**:

This script will plot the markers as small dots
onto the analyzed images. You have to provide validx.m,
validy.m, filenamelist and
the images in the ‘Current Folder’. After staring the
script you will be asked if you want to create a video or not. If you click
‘yes’ a folder ‘Video\_Markers’ will be created and
each frame captured as a \*.jpg file.

 

**Input and output
files:**

**Image files** should be 8 bit greyscale
Tiff (\*.tif) images and should be named with a
increasing number at the end.

If you want to use ‘filelist\_generator.m’,
the filename should be something like ‘PIC0’ or 
‘PIC1’ plus the number at the end scaling from ‘0001’ to  ‘9999’. The full name would be for the first
file ‘PIC10001.tif’. If you need to process more than 9999 image then you have
to modify ‘filelist\_generator.m’ or write an email to
us. The **‘filenamelist.mat’** is a matlab file since it was easier to combine text and numbers
into one file by just saving it in this format.

**‘time\_image.txt’** contains the time the image was
captured. Please keep in mind that using other software to change the name or
the format after capturing the images can lead to a change of the date and
capturing time of the images. It happens that the software will change the name
of the images and the new creation date and time of each image will be the time
it was renamed. Programs like Irfanview have the
option to preserve the original time of the images. This option has to be
checked to make sure you can match stress and strain at the end of your
analysis.

**‘grid\_x.dat’** and **‘grid\_y.dat’** are the files containing the x- and y-pixel position
of the starting grid created by the ‘grid\_generator.m’
function. If you want to create your own grids, you can do that with excel and
save them as tab delimited ASCII files. Both files can be organized as column
vectors or matrices, as long as they are equal.

**‘validx.dat’** and **‘vaildy.dat’** are both ASCII formatted tab delimited files which
contain in columns the position of each marker for each image.

**‘fitxy.dat’** will be only saved if you use ‘peak\_labelling.m’ or ‘pickpeak.m’
and contains all fitting parameters for each peak.

  

7.
Acknowledgement

 

Prof. W. N. Sharpe J. provided some helpful
hints what would be important to the user and what would be a waste of time
;-). I want to acknowledge him since it is always a pleasure to work in his lab
at the JHU.

We got  a lot of help from all our colleagues
off our near and far communities and we do appreciate this a lot. Please
comment on our Mathworks site if you need something or if you think this tool
works as it should.
