# Supplementary material for: Distinct roles of nonmuscle myosin II isoforms for establishing tension and elasticity during cell morphodynamics
Source: eLife. 2021 Aug 10;10:e71888. doi: 10.7554/eLife.71888 (PMC8391736; doi:10.7554/eLife.71888)
Supplement: Source data 2. [file elife-71888-data2.zip › Western Blots_raw images/Western Blot_Described.pptx]

## Slide 1
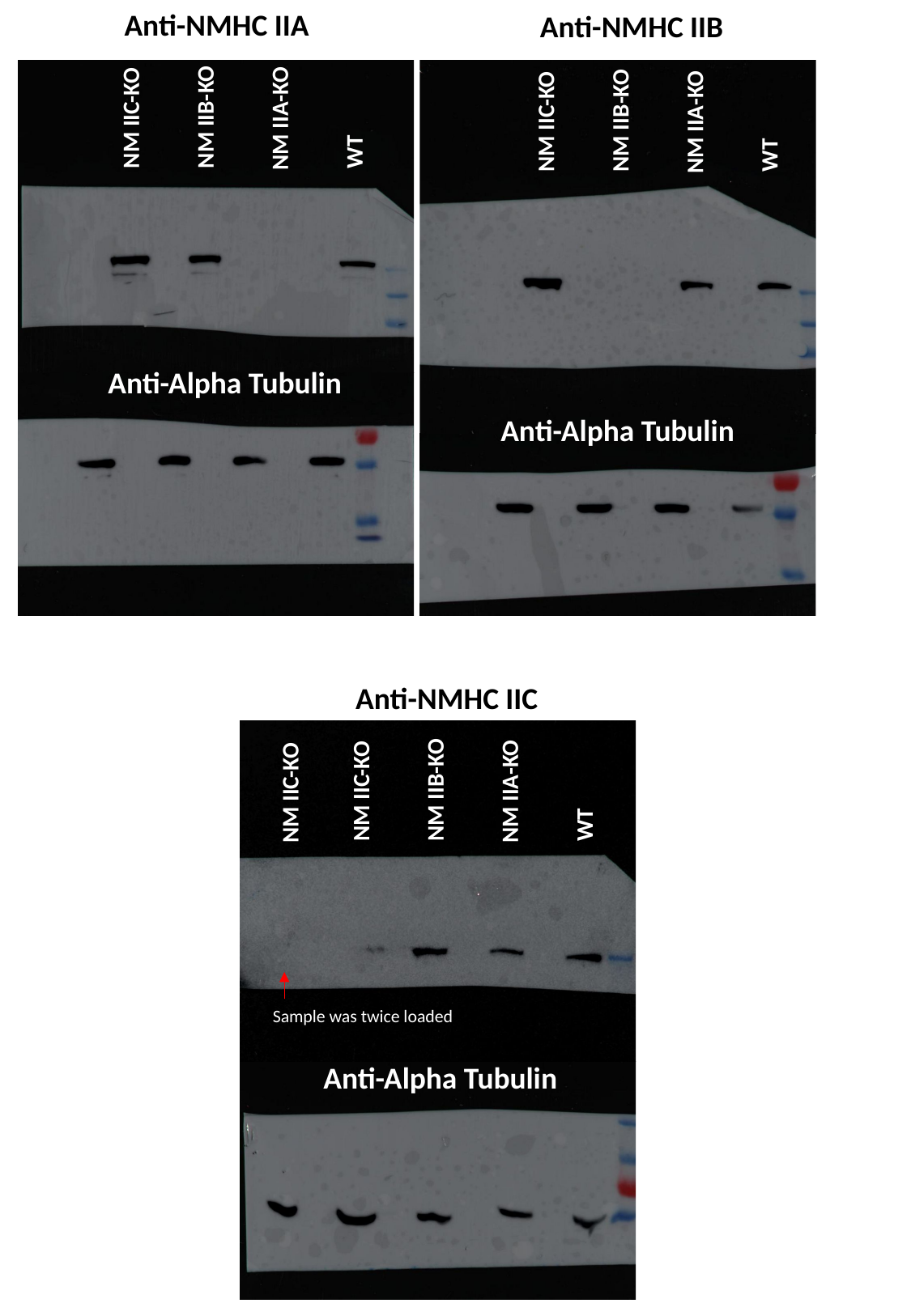

Anti-NMHC IIA
Anti-NMHC IIB
NM IIC-KO
NM IIB-KO
WT
NM IIA-KO
NM IIC-KO
NM IIB-KO
WT
NM IIA-KO
Anti-Alpha Tubulin
Anti-Alpha Tubulin
Anti-NMHC IIC
NM IIC-KO
NM IIB-KO
WT
NM IIC-KO
NM IIA-KO
Sample was twice loaded
Anti-Alpha Tubulin

## Slide 2
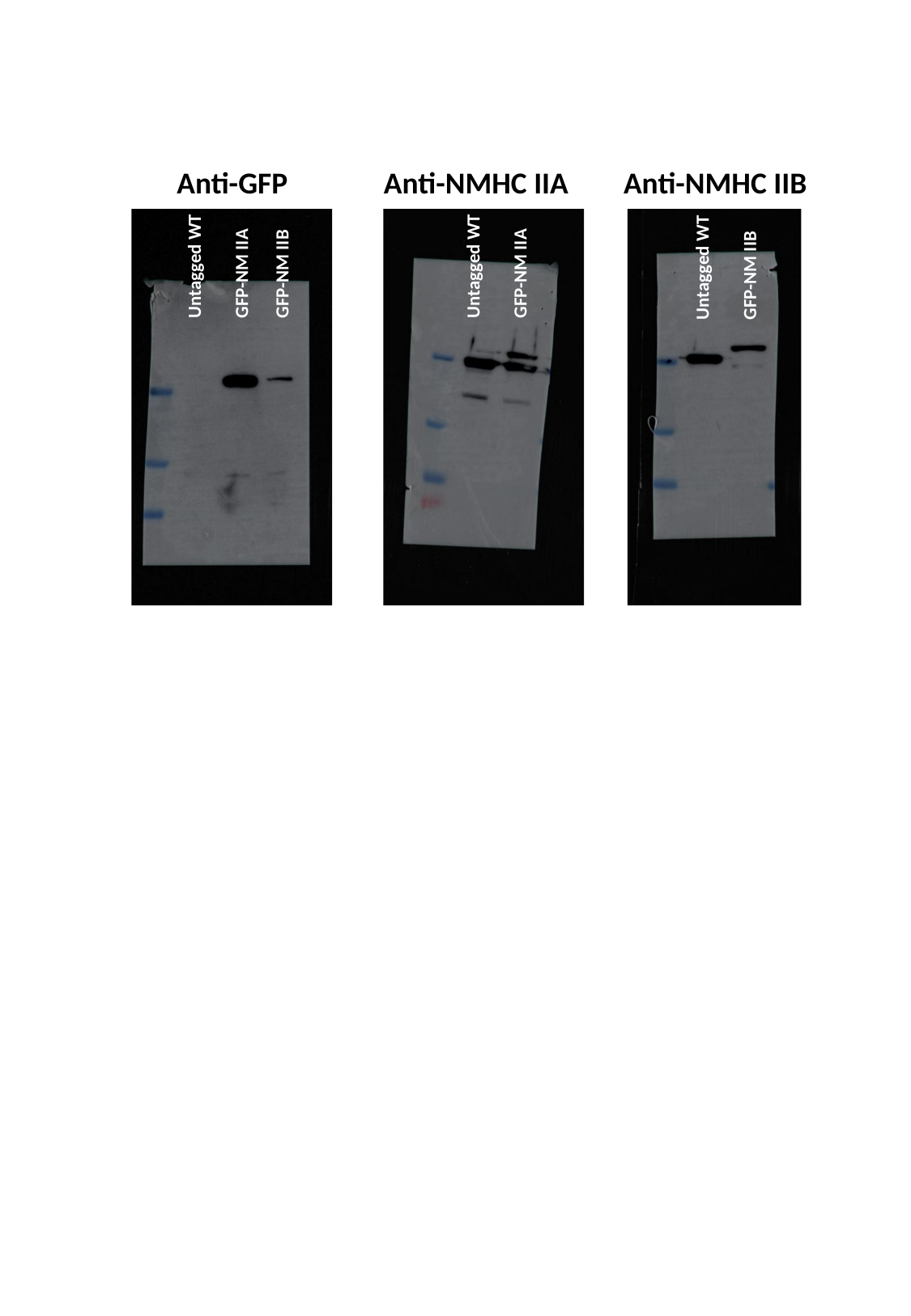

Anti-GFP
Anti-NMHC IIA
Anti-NMHC IIB
Untagged WT
GFP-NM IIA
Untagged WT
GFP-NM IIA
GFP-NM IIB
Untagged WT
GFP-NM IIB
